# Global identification of Smad2 and Eomesodermin targets in zebrafish identifies a conserved transcriptional network in mesendoderm and a novel role for Eomesodermin in repression of ectodermal gene expression

**DOI:** 10.1186/s12915-014-0081-5

**Published:** 2014-10-03

**Authors:** Andrew C Nelson, Stephen J Cutty, Marie Niini, Derek L Stemple, Paul Flicek, Corinne Houart, Ashley EE Bruce, Fiona C Wardle

**Affiliations:** Randall Division of Cell and Molecular Biophysics, New Hunt’s House, King’s College London, Guy’s Campus, London, SE1 1UL UK; MRC Centre for Developmental Neurobiology, New Hunt’s House, King’s College London, Guy’s Campus, London, SE1 1UL UK; Wellcome Trust Sanger Institute, Wellcome Trust Genome Campus, Hinxton, Cambridge, CB10 1HH UK; European Molecular Biology Laboratory, European Bioinformatics Institute, Wellcome Trust Genome Campus, Hinxton, Cambridge, CB10 1SD UK; Department of Cell and Systems Biology, University of Toronto, 25 Harbord Street, Toronto, ON M5S 3G5 Canada; Current address: Sir William Dunn School of Pathology, South Parks Road, Oxford, OX1 3RE UK

**Keywords:** Nodal, Smad2, Eomesodermin, Foxh1, Neural, Transcriptional regulation

## Abstract

**Background:**

Nodal signalling is an absolute requirement for normal mesoderm and endoderm formation in vertebrate embryos, yet the transcriptional networks acting directly downstream of Nodal and the extent to which they are conserved is largely unexplored, particularly *in vivo*. Eomesodermin also plays a role in patterning mesoderm and endoderm in vertebrates, but its mechanisms of action and how it interacts with the Nodal signalling pathway are still unclear.

**Results:**

Using a combination of expression analysis and chromatin immunoprecipitation with deep sequencing (ChIP-seq) we identify direct targets of Smad2, the effector of Nodal signalling in blastula stage zebrafish embryos, including many novel target genes. Through comparison of these data with published ChIP-seq data in human, mouse and *Xenopus* we show that the transcriptional network driven by Smad2 in mesoderm and endoderm is conserved in these vertebrate species. We also show that Smad2 and zebrafish Eomesodermin a (Eomesa) bind common genomic regions proximal to genes involved in mesoderm and endoderm formation, suggesting Eomesa forms a general component of the Smad2 signalling complex in zebrafish. Combinatorial perturbation of Eomesa and Smad2-interacting factor Foxh1 results in loss of both mesoderm and endoderm markers, confirming the role of Eomesa in endoderm formation and its functional interaction with Foxh1 for correct Nodal signalling. Finally, we uncover a novel role for Eomesa in repressing ectodermal genes in the early blastula.

**Conclusions:**

Our data demonstrate that evolutionarily conserved developmental functions of Nodal signalling occur through maintenance of the transcriptional network directed by Smad2. This network is modulated by Eomesa in zebrafish which acts to promote mesoderm and endoderm formation in combination with Nodal signalling, whilst Eomesa also opposes ectoderm gene expression. Eomesa, therefore, regulates the formation of all three germ layers in the early zebrafish embryo.

**Electronic supplementary material:**

The online version of this article (doi:10.1186/s12915-014-0081-5) contains supplementary material, which is available to authorized users.

## Background

Normal metazoan development occurs through the correct activation of different signalling pathways leading in turn to the temporally precise activation of transcriptional networks [1]. One such pathway with a fundamental and conserved role in early vertebrate development is the Nodal signalling pathway. Nodal signalling acts through ligand-mediated receptor activation of the transcription factors Smad2/3. On pathway activation these factors translocate to the nucleus where they interact with other transcription factors at genomic *cis*-regulatory elements to modulate target gene expression [2], leading to induction of mesoderm and endoderm, ventral neural tube formation and establishment of bilateral asymmetry [3]. The importance of this pathway in early development is seen in loss of Nodal signalling mutants in mouse and zebrafish, which display perturbed mesoderm, endoderm and ventral neural tube formation (for example, [4-8]). Similarly, in *Xenopus*, knockdown of Nodal signalling or dominant-negative interference with the pathway leads to inhibition of mesoderm and endoderm formation (for example, [9,10]). Equally, overexpression of Nodal signalling pathway components leads to upregulation of mesodermal and endodermal markers in zebrafish and *Xenopus* [11-13]. Despite this, the extent to which the transcriptional networks directed by Smad2 are evolutionarily maintained has not been determined.

In order to understand the transcriptional networks driven by Nodal signalling it is also necessary to unravel the functional relationship between Smad2/3 and their interacting factors. Since the initial identification of Foxh1 as a Smad-interacting transcription factor [14,15] several other transcription factors that interact with Smad2 in different systems have been identified, including E2A, HEB, Oct1 and Eomesodermin [15-19].

Eomesodermin is a T-box transcription factor expressed during early vertebrate development. It is critical for endoderm and cardiac mesoderm formation in mouse embryo [20,21], necessary and sufficient for mesoderm induction in *Xenopus* [22] and in differentiated human embryonic stem cells (ESCs) it has also been shown to act with Smad2/3 in the specification of endoderm [23].

In zebrafish there are two Eomesodermin homologues, *eomesa* and *eomesb* [24], with Eomesa being a maternally contributed factor that is not spatially restricted in early development [25,26]. Overexpression of Eomesa leads to induction of dorsal mesodermal markers and, in conjunction with Gata5 and Bon, Eomesa directly induces *sox32*, a key endodermal determinant [27-32]. On the other hand, MZ*eomesa* null mutant embryos exhibit only reduced early expression of endoderm markers and moderate lethality by 24 hours post fertilization [26], indicating that although Eomesa is sufficient for induction of mesoderm and endodermal genes it is not absolutely required for their expression. Redundancy of other interacting factors is likely to explain this, as in the case of Gata5 and Bon in *sox32* induction, and a recent study has suggested that Foxh1 and Eomesa act redundantly to mediate all Nodal signalling in the zebrafish embryo [33].

To understand better the transcriptional networks that operate in the early zebrafish embryo, we sought to characterize the earliest targets of Nodal signalling in zebrafish – just after the onset of zygotic transcription. Using a combination of Smad2 chromatin immunoprecipitation sequencing (ChIP-seq) and expression microarrays we identify direct targets of Nodal signalling. In addition, by reference to published human, mouse and *Xenopus* Smad2 ChIP-seq data we show that genomic binding in zebrafish and these species is highly predictive of Nodal-responsiveness, indicating that the transcriptional network directed by Smad2 is conserved in vertebrate mesendoderm.

We also examined the role of Eomesa in Nodal signalling using ChIP-seq for Eomesa. Our findings suggest that Eomesa is a general component of the Nodal transcriptional complex during mesendoderm formation. We also find that Eomesa acts combinatorially with Foxh1 in formation of endoderm and regulation of some mesodermal gene expression. Finally, we use RNA-seq analysis of MZ*eomesa* mutant embryos combined with our Eomesa ChIP-seq data and find that Eomesa also acts to repress inappropriate transcription of ectodermal genes. Thus, our data demonstrate a new role for Eomesa in early development and illustrates that the timing of developmental gene expression can be mediated by active repression as well as transcription initiation.

## Results

### Integrated genomic binding and expression data define a set of novel and known Nodal targets in zebrafish

**Figure 1:**
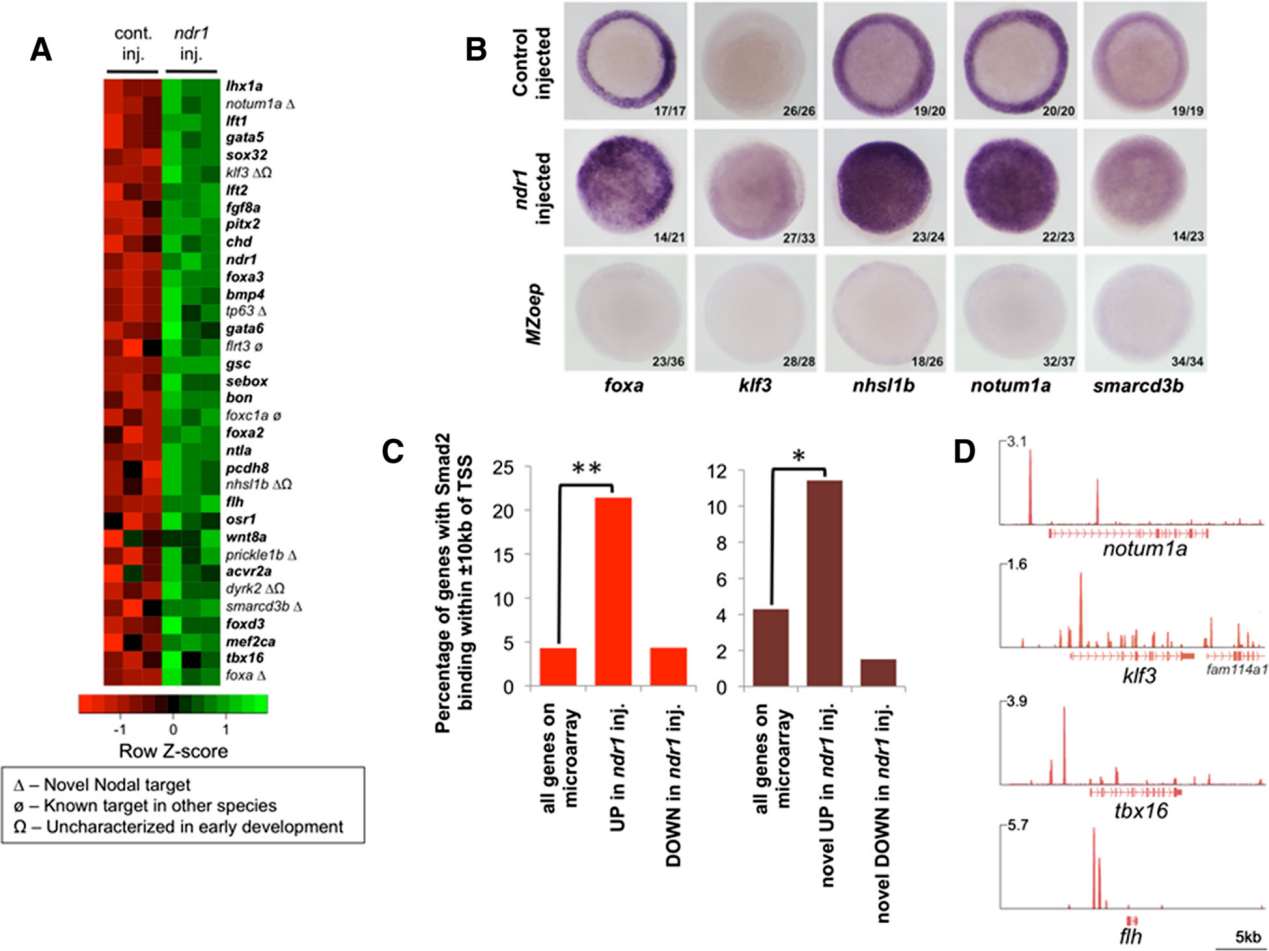
***ndr1*** **overexpression in zebrafish blastulas identifies known and novel Nodal target genes. (A)** Heatmap of a representative selection of genes induced on *ndr1* overexpression across the full range of *P* values and fold changes. Genes previously identified as Nodal target genes in zebrafish are in bold. **(B)** *In situ* hybridisation of wild type and *ndr1* mRNA-injected embryos for *foxa*, *klf3*, *nhsl1b*, *notum1a* and *smarcd3* at 50% epiboly showing upregulation in response to *ndr1* and absence in MZ*oep* mutant embryos that have no Nodal signaling. Animal views; dorsal to the right. Numbers on each panel indicate the number of embryos showing the phenotype depicted over the total number of embryos analysed. For *foxa* expression in MZ*oep* mutants the remaining 13/36 embryos showed absent expression except for a patch on one side of the embryo. For *nhsl1b* and *notum1a* the remaining 8/26 and 5/37 embryos, respectively, showed reduced expression around the margin. **(C)** Comparison of all genes represented on the microarray, or *ndr1*-responsive genes (either up- or down-regulated) to proximal Smad2 binding; comparison was performed for both all and novel *ndr1*-responsive genes. Compared to all genes on the microarray or those that are down-regulated in response to *ndr1*, up-regulated *ndr1*-responsive genes (both all and novel) are significantly associated with Smad2 binding. *P =7 × 10^−7^; **P =1 × 10^−40^. **(D)** Examples of Smad2 binding upstream of known (*tbx16* and *flh*) and novel targets; scale in reads per million reads.

In zebrafish, loss of Nodal signalling results in the loss of endoderm and most mesoderm, but despite both single gene and genome-scale approaches previously being used to identify Nodal target genes in zebrafish [17,34] the full range of Nodal target genes are unlikely to have been identified. In particular, targets at blastula stages have not been systematically studied, despite this being the time when Nodal signalling is required for specification of mesoderm and endoderm [35]. Thus, to identify additional novel Nodal targets at this critical time in development we performed microarray expression profiling of embryos at sphere stage (four hours post fertilization; hpf) injected with mRNA for the zebrafish Nodal homologue *nodal-related one* (*ndr1*). This revealed 252 upregulated and 69 downregulated genes compared to control embryos (*P* ≤0.02, Figure 1A; Additional file 1), which we henceforth refer to as Ndr1-responsive genes. Comparison with published data revealed that 200 upregulated and 66 downregulated genes had not previously been implicated as Nodal target genes (see Additional file 1: Table S1 and Additional file 2: Figure S1).

We next sought to validate these results on a selection of the novel targets: *foxa*, *klf3*, *nhsl1b*, *notum1a* and *smarcd3b* by *in situ* hybridisation at sphere and 50% epiboly (5.3 hpf). At sphere stage we were able to detect an increase in expression of *foxa*, *klf3*, *nhsl1b* and *notum1a* in *ndr1* mRNA-injected embryos compared to control embryos (see Additional file 2: Figure S1), even though *in situ* hybridisation is a less sensitive technique than microarray. Conversely, we did not detect expression of these genes in maternal-zygotic *oep* (MZ*oep*) mutant embryos, which have no Nodal signaling [36] (see Additional file 2: Figure S1). By 50% epiboly we were able to detect an increase in expression of all five genes by *in situ* hybridisation in *ndr1*-injected embryos, whilst MZ*oep* mutant embryos had reduced or absent expression (Figure 1B). These results validate our microarray data and indicate that these genes are targets of Nodal signalling.

We next performed ChIP-seq using an antibody specific for Smad2 (see Additional file 3: Figure S2) in wild-type embryos at high (3.3 hpf)-sphere stage and integrated this with our overexpression data to identify functional direct targets of Nodal signalling. This experiment revealed Smad2 binding at 898 regions across the whole genome (see Additional file 4: Table S2). To validate our ChIP-seq results we performed ChIP-qPCR on independent biological samples for a selection of regions with variable enrichment (see Methods). As shown in Figure S3 (see Additional file 5: Figure S3A) six of the seven peaks we tested have robust enrichment (from 3.1 to 8.2 fold) over the control immunoglobulin G (IgG) ChIP, whilst one peak, Smad2 peak 10, which was classified at ‘low enrichment’ in our ChIP-seq data (see Methods), did not show enrichment over IgG, showing our ChIP-seq data are concordant with independent ChIP-qPCR data.

Our analysis of the ChIP-seq data shows that Smad2 binding is enriched within ±10 kb of transcription start sites (TSSs; Additional file 6: Figure S4A; henceforth referred to as proximal binding), revealing a total of 729 zebrafish genes with Smad2 proximal binding (see Additional file 4: Table S2). When considering Ndr-1 responsive genes we also find Smad2 binding is enriched within ±10 kb of their TSSs (see Additional file 6: Figure S4B). From this analysis we find 53 genes that are Ndr1-responsive and have proximal Smad2 binding, suggesting they are direct targets of Smad2-mediated Nodal signalling. Of those, 30 were previously known to be Nodal-regulated including genes required for endoderm formation, such as *bon* and *sox32*, and genes required for correct mesoderm formation, such as *ntla, tbx16*, *fgf8a, chd and flh* (see Additional file 4: Table S2). These known targets are significantly associated with Smad2 binding (Figure 1C), as are our novel Ndr1-responsive genes (Figure 1C), reinforcing the functional significance of Smad2 proximal binding and suggesting we have identified *bona fide* direct targets of Smad2. Examples of Smad2 binding proximal to known and novel target genes is shown in Figure 1D. Furthermore, the novel targets we identified include genes that have roles consistent with Nodal activity, such as *notum1a* and *smarcd3b*, which have roles in head formation through Wnt antagonism [37] and establishment of left-right asymmetry [38], respectively. These novel targets will be of interest in future studies of Nodal signalling.

### The transcriptional network directed by Smad2 is conserved amongst vertebrates

Although the Nodal signalling pathway has been well studied over the last 20 years in multiple vertebrate systems, the extent to which the downstream transcriptional network directed by Nodal signalling is conserved has not been comprehensively assessed. Our datasets combined with the recently generated human, mouse and *Xenopus* Smad2 ChIP-seq datasets [19,39,40] offer the first opportunity to explore this, and we sought to exploit these data in order to identify conserved Smad2 targets in all vertebrates. The mammalian datasets are derived from treated ESCs which are likely to mimic some of the conditions of the zebrafish blastula including gradients of Nodal signalling. More specifically, Kim and colleagues performed ChIP-seq for Smad2/3 in control human ESCs and ESCs differentiated to endoderm [39] while Lee and colleagues performed ChIP-seq for Smad2 in control mouse ESCs, and ESCs treated with either Activin or a Nodal/Activin receptor inhibitor [40]. The *Xenopus* dataset represents Smad2/3 binding in whole gastrulae [19].

**Figure 2:**
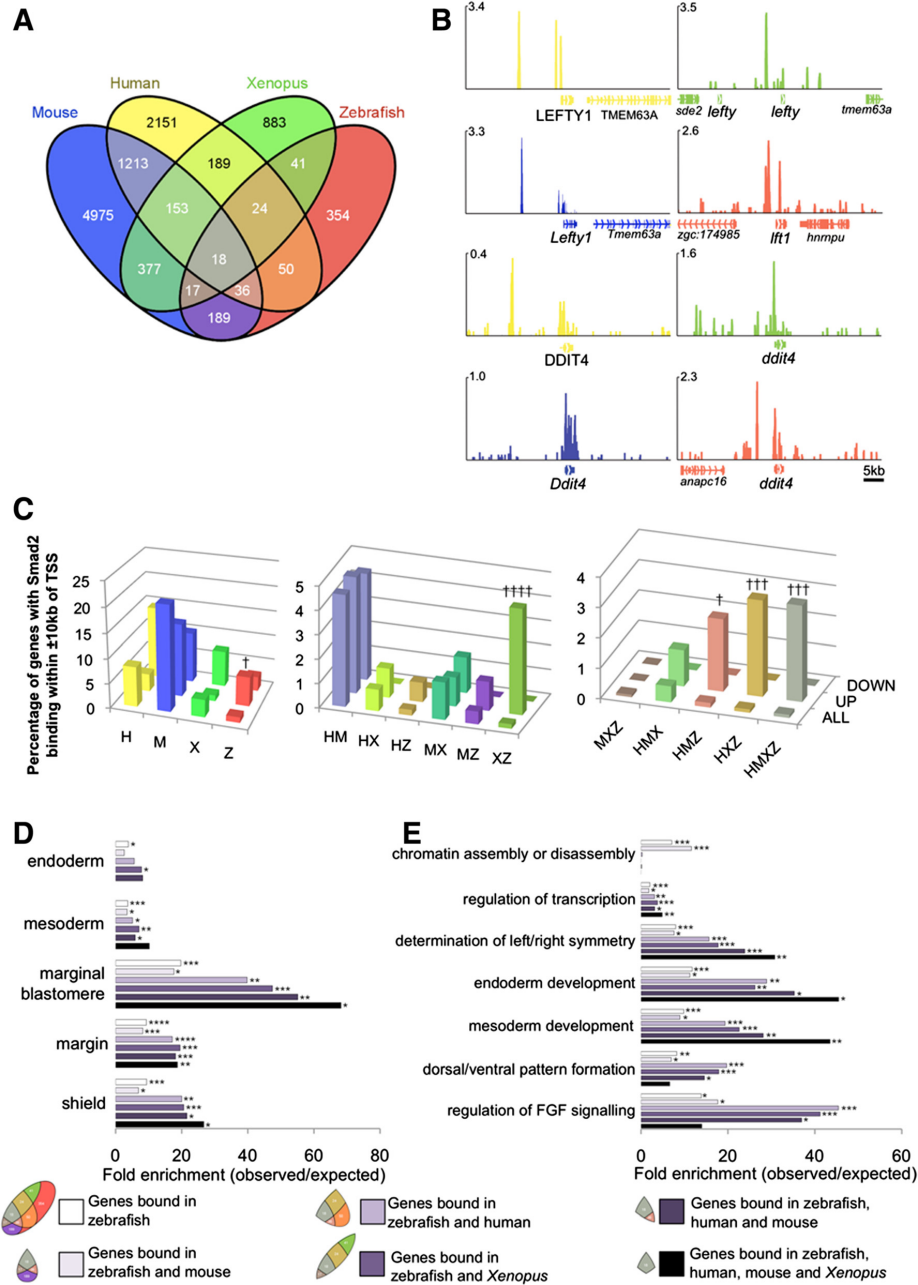
**The transcriptional network directed by Nodal signalling is substantially conserved in vetebrates. (A)** Venn diagram indicating the number and overlap of genes with Smad2 binding ±10 kb of TSSs in ChIP-seq datasets for each of four vertebrates. **(B)** Examples of genes with proximal Smad2 binding in all four species; scale in reads per million reads. Colour coded as in A. **(C)** Relationship between *ndr1*-responsive genes and proximal Smad2 binding in 1 (left), 2 (middle) or 3+ species (right). Colour coded as in A. †*P* ≤1 × 10^−12^; †††*P* ≤1 × 10^−42^; ††††*P* ≤1 × 10^−48^. **(D)** Fold enrichment for genes expressed at sites of Nodal activity amongst genes with proximal Smad2 binding in zebrafish, or zebrafish and other species. **(E)** Fold enrichment for genes involved in known Nodal-mediated processes amongst genes with proximal Smad2 binding in zebrafish, or zebrafish and other species. Key defined as gene subsets as in A. *P ≤5 × 10^−2^; **P ≤3 × 10^−4^; ***P ≤5 × 10^−6^; ****P ≤1 × 10^−20^. ChIP-seq, chromatin immunoprecipitation sequencing; TSSs, transcription start sites.

In order to draw a comparison of genes with proximal Smad2 binding in these other vertebrates with binding in zebrafish, mammalian and *Xenopus* genes were converted to orthologous zebrafish gene IDs (see Methods). Genes with proximal Smad2 binding within ±10 kb of their TSSs were then compared. Notably, genes with proximal binding in each species exhibited considerable overlap, with 51% (375/729) of genes bound in zebrafish also exhibiting proximal binding in at least one mammalian species (Figure 2A; Additional file 7: Table S3). Representative peak images of genes with Smad2 binding in multiple species are shown in Figure 2B, including the known Nodal target gene, *lft1*, and a hitherto unrecognized target and regulator of dorsoventral patterning, *ddit4* [41].

To determine whether this conserved binding may be functional we next compared genes with proximal Smad2 binding in all four species to the Ndr1-responsive genes from our zebrafish microarray experiment. This revealed that upregulated genes are more likely to have proximal binding in (1) all four species; (2) zebrafish and both mammals; (3) zebrafish, human and *Xenopus*; (4) zebrafish and *Xenopus*; and (5) zebrafish alone (Figure 2C). On the other hand, genes exhibiting proximal binding in mouse and/or human alone or *Xenopus* alone are no more likely to be Ndr1-responsive than by chance. These results suggest that the functional network directed by Smad2 in zebrafish is substantially conserved amongst other vertebrates.

We next asked where genes with proximal Smad2 binding are expressed and what functions they perform in zebrafish (Figure 2D-E). As expected, genes with proximal binding in zebrafish show enriched expression at the principle sites of Nodal activity in early zebrafish embryogenesis, such as the margin and shield (Figure 2D). Interestingly though, genes with proximal binding in zebrafish and one or more other species show even greater enrichment for these tissues (Figure 2D) whilst genes exhibiting binding in both mouse and human but not zebrafish do not show such enrichment (see Additional file 8: Table S4). Similarly, genes with proximal binding in zebrafish and one or more other species show greater enrichment for genes involved in developmental processes known to be mediated by Nodal signalling, such as mesoderm and endoderm development (Figure 2E). In contrast, genes with proximal binding in mouse and human but not zebrafish are not enriched for known Nodal processes (see Additional file 8: Table S4). Taken together these results indicate that the conserved network directs mesendoderm formation.

Smad2 has been shown recently in mouse to regulate expression of chromatin assembly genes such as histones [42]. Notably, we detect enrichment for Smad2 binding proximal to such genes in zebrafish and mouse suggesting Smad2 may also play a role in regulating chromatin assembly genes in zebrafish (Figure 2E).

Thus, our analysis indicates that the functional network driven by Smad2 downstream of Nodal signalling is conserved in vertebrate embryos and the mechanisms by which Nodal directs mesendoderm formation in vertebrates is likely to be through regulation of the same target genes. In identifying these conserved targets our data represent a resource providing candidates for further study in the context of mesendoderm formation in vertebrates.

### Genomic co-occurrence of Smad2 and Eomesa is associated with Ndr1-responsive genes

**Figure 3:**
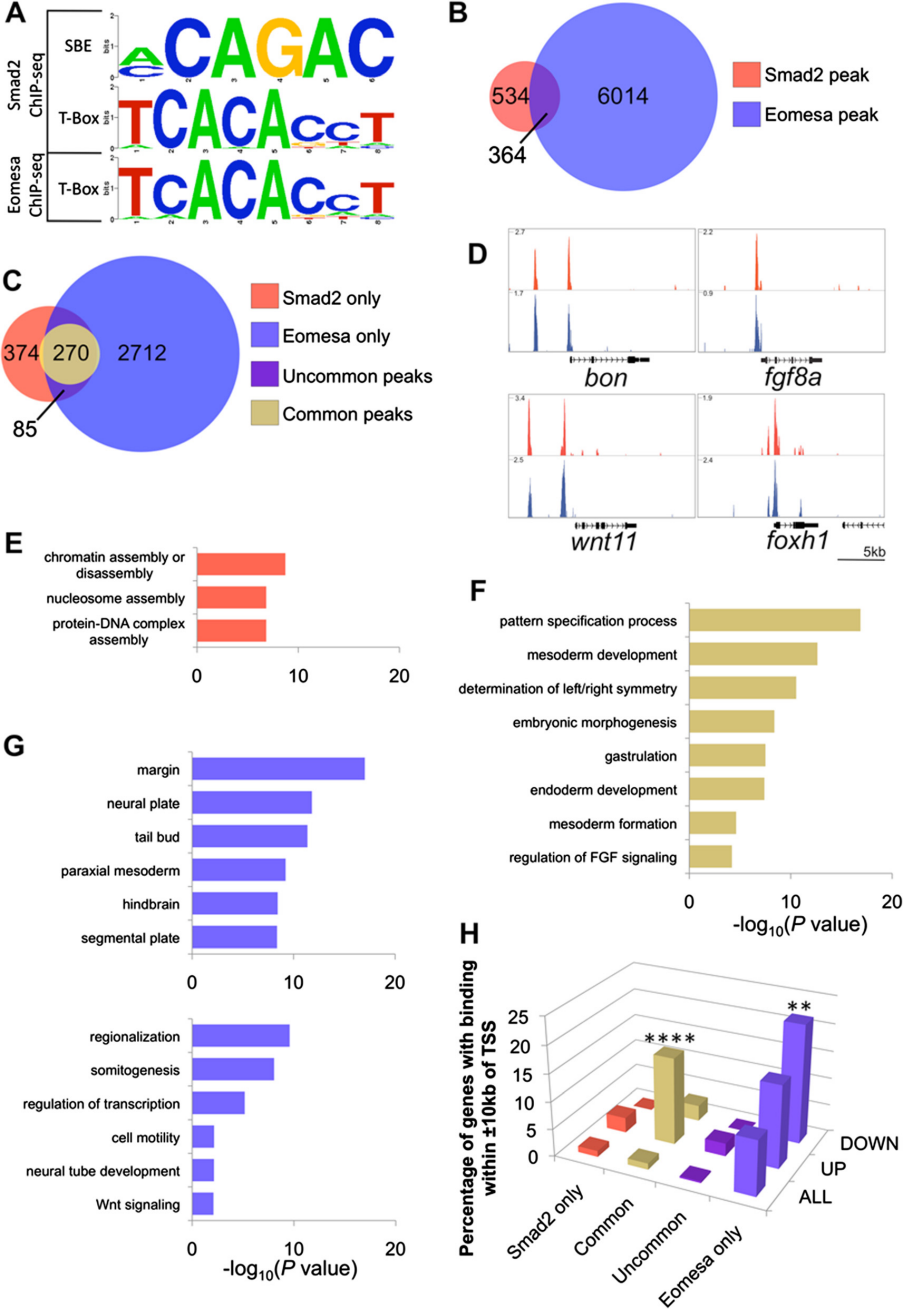
**Smad2 and Eomesa bind common regulatory elements proximal to** ***ndr1*** **-responsive genes and regulate the developmental functions of Nodal signalling. (A)** *De novo* motif analysis identifies the known Smad binding element (SBE) and consensus T-box binding site within Smad2 ChIP-seq peaks and Eomesa ChIP-seq peaks. **(B)** Venn diagram indicating the overlap between Smad2 and Eomesa ChIP-seq peaks. **(C)** Venn diagram of the overlap between genes with proximal Smad2 and Eomesa binding (within ±10 kb of their TSS). **(D)** Examples of genes with common proximal peaks of Smad2 and Eomesa binding; scale in reads per million reads. **(E-G)** Functional and anatomical analysis of genes showing Smad2 and/or Eomesa binding. Colour coded as in C. **(H)** Comparison of *ndr1*-responsive genes with subsets of genes with proximal Smad2 and/or Eomesa binding as defined in C. ** *P* ≤2 × 10^−3^; **** *P* ≤1 × 10^−105^. ChIP-seq, chromatin immunoprecipitation sequencing; TSS, transcription start site.

As an additional validation of our zebrafish Smad2 ChIP-seq data [43] we performed *de novo* motif finding for Smad2 bound regions and found the known Smad binding element [44] to be enriched and central to peaks (Figure 3A and Additional file 9: Figure S5), indicating we have identified *bona fide* targets of Smad2. Interestingly, we also found the consensus T-box binding site (TBS; [45] to be highly enriched in these regions (Figure 3A), suggesting Smad2 may act with a T-box transcription factor in regulating Nodal gene expression. In this respect Eomesodermin is an obvious candidate: it has an evolutionarily conserved role in mediating Nodal signalling, including through physical interaction with Smad2 [18,21,23,33] and in zebrafish Eomesodermin homologue A (Eomesa) is maternally contributed and expressed in the early embryo [25,26]. Furthermore induction of some Eomesa target genes in zebrafish gastrulae is Nodal-dependent [25,46]. In order to explore this further we performed ChIP-seq in zebrafish using a polyclonal antibody specific for Eomesa (see Additional file 3: Figure S2) at the same stage as our Smad2 ChIP. This revealed 6,378 genomic binding regions for Eomesa and 3,066 genes with proximal Eomesa binding within 10 kb of their TSS (see Additional file 10: Table S5). In order to validate our ChIP-seq data we performed ChIP-qPCR on independent biological samples for a selection of peaks that are either bound by Eomesa alone or have common binding with Smad2 (see Methods for further details). As shown in Figure S3 (see Additional file 5: Figure S3B) six of the seven peaks we tested, including all those that are common peaks, have robust enrichment (from 5.3-25.8 fold) over control IgG ChIP, again showing concordance of our ChIP-seq results with independent ChIP-qPCR data. As further validaton, *de novo* motif finding identified the TBS as enriched and central to peaks in Eomesa bound regions (Figure 3A and Additional file 9: Figure S5).

We next asked if Eomesa and Smad2 binding co-occurs and found considerable overlap in their genomic binding coordinates, with 41% (364/898) of all Smad2 bound regions co-occurring with Eomesa (Figure 3B,D; Additional file 11: Table S6), validating our previous observation that Smad2 bound regions are enriched for the consensus TBS. Of genes with proximal Smad2 binding, 37% (270/729) also exhibit Eomesa binding at the same coordinates (referred to as ‘common’ peaks; Figure 3D and Additional file 11: Table S6), while a further 12% (85/729) show independent binding of Smad2 and Eomesa in the ±10 kb proximal region (referred to as ‘uncommon’ peaks). However, T-box and Smad binding elements do not form part of the same motif, and although they appear in close proximity to each other they have inconsistent spacing, orientation and strand orientation relative to each other. We also note that of the genes with both Smad2 and Eomesa proximal binding, a high percentage, 54% (191/355), show proximal Smad2 binding in at least one species other than zebrafish (see Additional file 7: Table S3). These results suggest that Eomesa may mediate the output of Nodal signalling at the level of transcription through interaction with Smad2, and that the common target genes are conserved Nodal target genes in mesendoderm formation.

We next performed Gene Ontology (GO) and anatomical term analysis of genes with proximal binding and find that lone Smad2 binding is associated with chromatin genes as previously noted (Figure 3E; Additional file 8: Table S4), whilst lone Eomesa binding is associated with somitogenesis and neural genes (Figure 3F; Additional file 8: Table S4). We also observe that, consistent with our observations above, common binding is associated with the early developmental functions of Nodal signalling in mesendoderm formation and embryonic patterning (Figure 3G; Additional file 8: Table S4).

In an effort to understand if this common binding is functional we next tested how proximal Eomesa and Smad2 binding relates to our Ndr1-responsive genes. We found that upregulated genes are significantly associated with common Eomesa and Smad2 binding in zebrafish whereas proximal binding of Smad2 alone is not (Figure 3H). Significantly, functional and anatomical analysis reveals that Ndr1-responsive genes with common proximal binding are associated with Nodal developmental functions and sites of Nodal action, whereas those with proximal binding of Smad2 without Eomesa are not (see Additional file 8: Table S4), again indicating that a large part of Smad2 signalling in zebrafish blastulae involves Eomesa. Furthermore, genes with proximal Smad2 and Eomesa binding in zebrafish, as well as Smad2 binding in at least one other species are enriched for developmental functions whilst genes having only proximal Smad2 in zebrafish and at least one other species, show no such enrichment (see Additional file 8: Table S4). Taken together these data suggest that Eomesa participates in the conserved developmental functions of Nodal signalling by regulating a conserved transcriptional network.

As previously noted, lone Eomesa binding is associated with genes expressed in structures such as the neural plate (Figure 3F). Our analysis also revealed a set of genes downregulated on *ndr1* injection that are significantly associated with lone Eomesa genomic binding (Figure 3H), which are enriched for known expression in neural structures (see Additional file 8: Table S4). However since a consequence of ectopic *ndr1* expression is induction of mesendoderm leading to a reduction of ectoderm, this association may be due to loss of ectoderm on *ndr1* overexpression. Nevertheless, since Eomesa binding is associated with genes expressed in neural structures (Figure 3F), it could also imply Eomesa has a role in regulating ectodermal gene expression in zebrafish blastulae and we explore this later.

Taken together our data indicate that Smad2 and Eomesa act together to regulate early Nodal activity, suggesting Eomesa may be a general component of the Nodal transcriptional complex in the zebrafish blastula. Moreover, Smad2 and Eomesa may have independent roles in regulating chromatin assembly and ectodermal gene expression, respectively.

### The combinatorial activity of Eomesa and Foxh1 is required for endoderm formation and some mesodermal gene expression

Our data show that Eomesa and Smad2 bind proximally to genes required for mesoderm and endoderm formation such as *ntla* and *bon*; however, we previously showed that mesoderm formation and marker gene expression are only partially deficient in MZ*eomesa* mutants [26]. Recent RT-PCR analysis of MZ*eomesa* embryos, showed that *ntla* and *gsc* transcript levels are reduced at 30% epiboly, although they show some degree of recovery by shield stage (Xu *et al*., [46]). Taken together, this suggests that other factors may also be redundantly required with Eomesa to regulate robust mesodermal gene expression. Foxh1 is a known Smad2 interacting factor that mediates Nodal signalling and a recent study by Slagle *et al*. [33] has implicated Eomesa in mediating mesendodermal gene expression in combination with Foxh1. In addition, in human ESCs Smad2/3 genomic occupancy is almost completely coincident with Foxh1 [39]. Thus, we attempted to characterize Foxh1 binding in zebrafish blastulae to further explore the relationship between Smad2, Eomesa and Foxh1. Unfortunately, no appropriate ChIP-grade antibody against zebrafish Foxh1 exists to our knowledge and exhaustive attempts to perform ChIP-seq for Foxh1 have been unsuccessful. The conserved mammalian and amphibian Foxh1 genomic binding consensus has been well characterized, however [19,47]. We used the position frequency matrix available in the JASPAR database [48] to identify potential Foxh1 binding sites in zebrafish. From this analysis we found that consensus Foxh1 binding sites were present in 24% and 43% of Eomesa and Smad2 ChIP-seq peaks, respectively, but occurred in 51% of common Smad2/Eomesa ChIP-seq peaks (see Additional file 9: Figure S5 and Additional files 4, 10 and 12). This is a significant enrichment (*P* value =5.7 × 10^−30^ compared to Eomesa peaks and 4.9 × 10^−3^ compared to Smad2 peaks) and implies that a functional relationship between Eomesa and Foxh1 exists through co-occurrence at a subset of Smad2-responsive regulatory elements. We, therefore, sought to explore the relative contribution of Eomesa and Foxh1 to early Nodal signalling in the zebrafish blastula.

**Figure 4:**
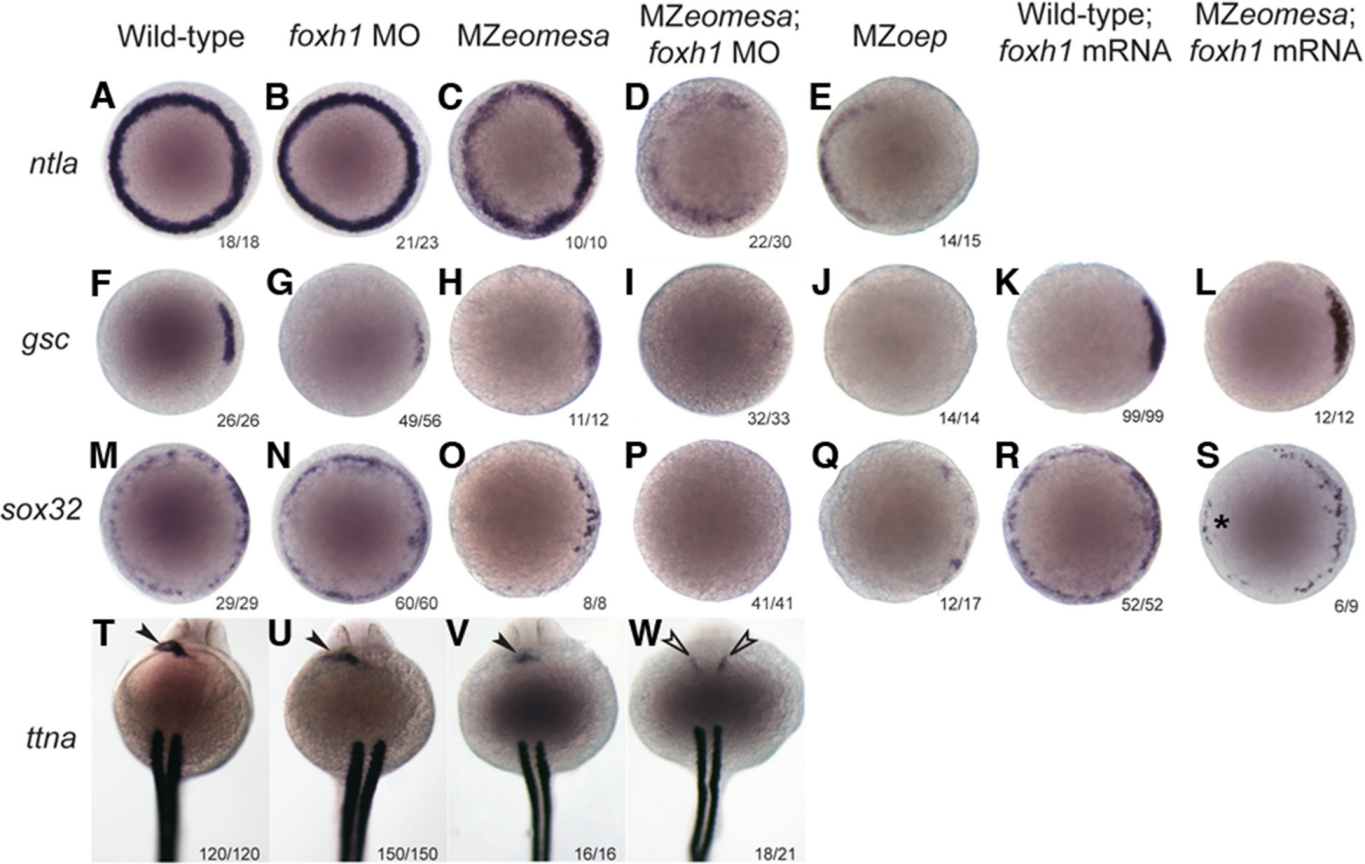
**Eomesa and Foxh1 combinatorially regulate early mesendoderm marker expression and are required for endoderm formation.** *In situ* hybridisation of *ntla* **(A-E)**, *gsc* **(F-L)** and *sox32* **(M-S)** at 30% epiboly in wild-type embryos, *foxh1* morphants, MZ*eomesa* mutants, MZ*eomesa*;*foxh1* morphants, MZ*oep* mutants, and in wild type embryos and MZ*eomesa* mutants injected with foxh1 mRNA. Rescued expression of sox32 expression in the ventral margin of MZ*eomesa* mutants injected with foxh1 mRNA is indicated by an asterisk*. In situ* hybridisation of *ttna* **(T-W)** at 24 hpf in wild-type embryos, *foxh1* morphants, MZ*eomesa* mutants and MZ*eomesa*;*foxh1* morphants showing expression in the heart (arrow heads) and somites. The open arrow heads indicate cardia bifida. Animal views; dorsal to the right. Numbers on each panel indicate the number of embryos showing the phenotype depicted over the total number of embryos analysed. For *sox32* expression in MZ*oep* mutants faint staining in the YSL was detected in 11/15 embryos as previously reported [54]. hpf, hours post fertilization.

In their recent study Slagle and colleagues [33] utilized *MZmid* mutants (*foxh1* mutants) with overexpression of *eomesa-eng* (Eomesa DNA binding domain fused to the engrailed transcriptional repressor domain) to assess the requirement for Eomesa and Foxh1 in Nodal signalling in mesendoderm. Here, we utilized a complementary approach, morpholino knock down of Foxh1 in MZ*eomesa* mutant embryos. However, whilst the *foxh1* morphants largely recapitulate the null phenotype of other animals [33,49-52], they do not exhibit notochord defects (data not shown), suggesting that Foxh1 knockdown is incomplete. Nevertheless, we performed *in situ* hybridisation of markers indicative of Nodal activity in the mesoderm (*ntla* and *gsc*) and endoderm (*sox32*) at 30% epiboly (4.7 hpf). Expression of *ntla* is not affected in *foxh1* morphants and is only mildly affected in MZ*eomesa* embryos; however, expression is substantially further downregulated, likely on the dorsal side, in MZ*eomesa* embryos injected with *foxh1* morpholino (MZ*eomesa;foxh1* morphants) suggesting both factors act combinatorially to regulate *ntla* expression at this stage (Figure 4A-D). Similarly, both factors are required together for *gsc* expression in the dorsal mesoderm (compare with Figures 4F-I), but consistent with notochord formation being unaffected, they are not required for *flh* expression (data not shown). Expression of the endoderm marker *sox32* is not greatly affected in *foxh1* morphants although it is downregulated in the ventral-lateral margin in MZ*eomesa* embryos; however, expression is completely absent in MZ*eomesa*;*foxh1* morphants, again indicating a combinatorial role for these factors (Figure 4M-P). The expression of *ntla, gsc* and *sox32* in the MZ*eomesa;foxh1* morphants at 30% epiboly is similar to that seen in MZ*oep* mutants (Figure 4E,J,Q), which have no Nodal signaling, suggesting that Eomesa and FoxH1 are together responsible for mediating the effects of Nodal signaling on expression of these genes. Although early expression of *ntla*, *gsc* and *sox32* at 30% epiboly is downregulated or absent in MZ*eomesa*;*foxh1* morphants, it is possible expression may recover at later stages; therefore, we analysed expression at shield stage (6 hpf; Additional file 12: Figure S6). Interestingly we found that *ntla* expression recovered in the dorsal, and to a variable extent in the ventral-lateral, margin, whilst *gsc* expression recovered to near normal in MZ*eomesa*;*foxh1* morphants. On the other hand, *sox32* expression continued to be strongly affected at shield stage, showing some recovery on the dorsal side but little recovery on the ventral-lateral side in MZ*eomesa*;*foxh1* morphants. Furthermore, consistent with this reduction in *sox32* expression at earlier stages, *in situ* hybridisation at 24 hpf for *ttna*, a marker of cardiac mesoderm and somite boundaries, shows the majority of MZ*eomesa*;*foxh1* morphants have cardia bifida (Figure 4W; open arrow heads), confirming an endoderm defect since heart precursors cells to not fuse at the midline in the absence of endoderm [29,53,54].

Finally we asked to what extent Foxh1 and Eomesa compensate for each other in regulating Nodal signaling by injecting *foxh1* mRNA into MZ*eomesa* mutant embryos, with the expectation that gene expression would be rescued if Foxh1 can substitute for Eomesa. When assaying for *gsc* expression, which is not much affected in MZ*eomesa* embryos, we did not see any change in expression when *foxh1* is overexpressed (Figure 4H,K-L); however, we found that *foxh1* mRNA is able to partially rescue expression of *sox32* in the ventral-lateral margin of MZ*eomesa* mutant embryos (Figure 4O, R-S; asterisk), suggesting FoxH1 acts redundantly with Eomesa in regulating *sox32* expression.

From these data we conclude that Eomesa and Foxh1 are together required for some early mesodermal gene expression, and for *sox32* expression and subsequent endoderm formation. However since the *foxh1* morpholino may result in incomplete Foxh1 knockdown it remains an open question, as to exactly which other targets of Nodal signalling are functionally regulated by both Eomesa and Foxh1. In order to definitely determine this it will be necessary to generate homozygous double maternal zygotic mutants for *eomesa* and *foxh1* (*midway* mutation). Efforts to do this are ongoing but have so far been unsuccessful.

### Eomesa represses ectodermal gene expression in the blastula

**Figure 5:**
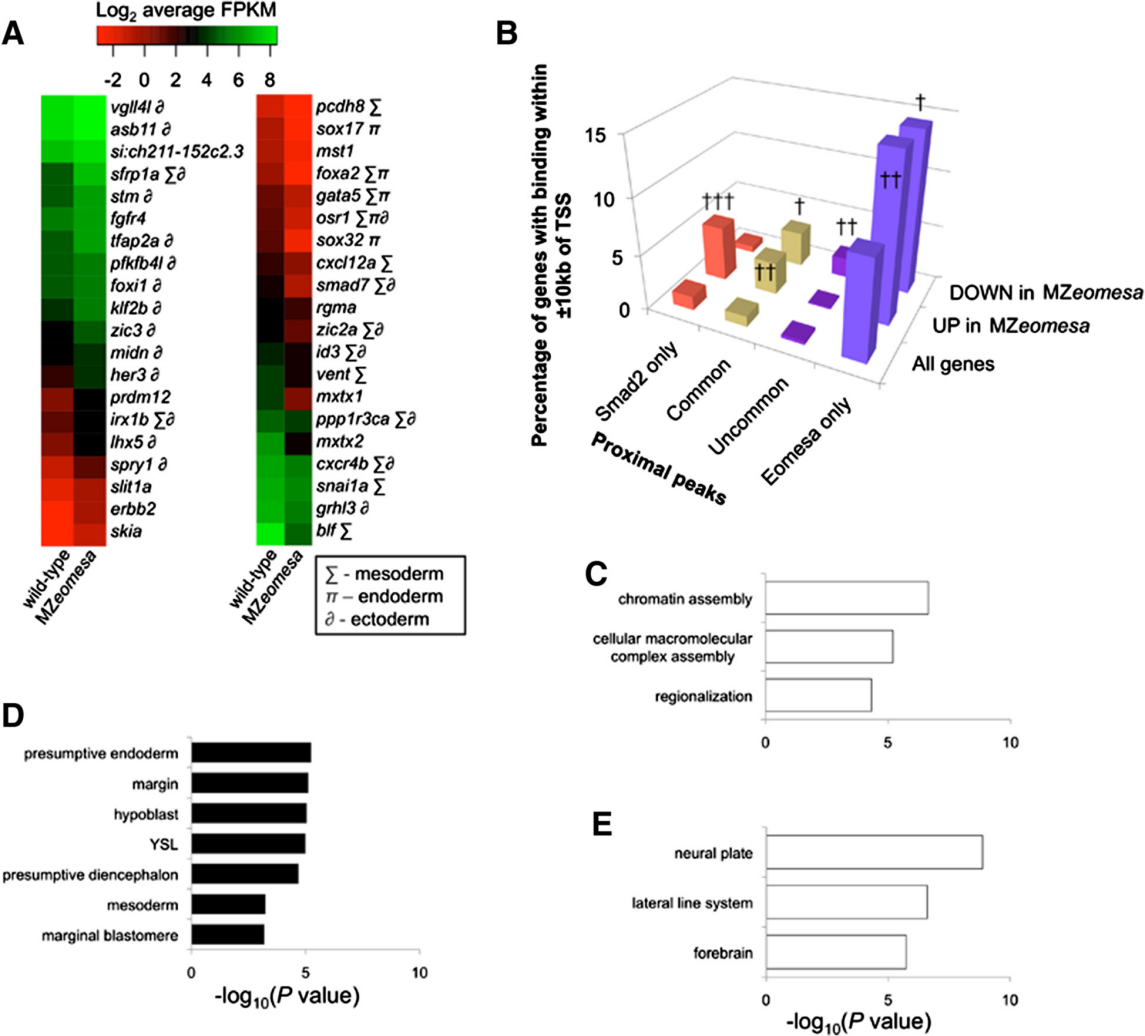
**Eomesa positively regulates mesendoderm and negatively regulates ectoderm markers and chromatin assembly genes. (A)** Heatmap of expression differences between wild-type and MZ*eomesa* mutants at sphere stage; π – endoderm; ∑ - mesoderm; ∂ - ectoderm. **(B)** Comparison of all genes, or those up- or down-regulated in MZ*eomesa* embryos compared to wild type with genes that have Smad2 only, common, uncommon or Eomesa only binding within ±10 kb of their TSSs (colour coded as in Figure 3C). Compared to all genes, those that are up- or down-regulated in MZ*eomesa* embryos are significantly associated with lone Eomesa binding. Genes that have common and uncommon binding are associated with down-regulated genes, whilst Smad2 only and common binding is associated with up-regulated genes in MZ*eomesa* embryos. † *P* ≤2 × 10^−5^; †† *P* ≤1 × 10^−7^; ††† *P* ≤1 × 10^−18^. **(C)** Functional annotation analysis of genes upregulated in MZ*eomesa* mutants. **(D)** Anatomical analysis of genes downregulated in MZ*eomesa* mutants. **(E)** Anatomical analysis of genes upregulated in MZ*eomesa* mutants. TSSs, transcription start sites.

Our Eomesa ChIP-seq data revealed 6,014 binding sites without significant detection of Smad2 at the same sites (Figure 3B and Additional file 10: Table S5), and functional GO term analysis indicates that genes with proximal binding of Eomesa without Smad2 have a potential role in neurogenesis and somitogenesis (Figure 3G and Additional file 8: Table S4). These functional roles, however, occur at a time point later than when our data were collected. Thus, in order to explore whether Eomesa is binding and regulating genes represented in these categories in the zebrafish blastula we generated RNA-seq data from wild-type and MZ*eomesa* mutant embryos at the same stage as our ChIP-seq data to identify Eomesa-responsive genes. This revealed 693 upregulated and 491 downregulated genes in MZ*eomesa* mutants (*P* ≤0.05; Figure 5A and Additional file 13: Table S7). Genes upregulated in MZ*eomesa* mutants are enriched for expression in the ectoderm, whilst downregulated genes are enriched for expression in mesendoderm (Figure 5A; Additional file 8: Table S4). This further suggests that Eomesa may be an activator in the context of mesendoderm and a repressor in the context of ectoderm.

We then compared these Eomesa-responsive genes to Eomesa and Smad2 genomic binding to identify functional binding. We find that lone Eomesa binding is significantly associated with genes both up- and down-regulated on loss of Eomesa, indicating that Eomesa binding independent of Smad2 is indeed functional and acts in both an active and repressive manner (Figure 5B). Interestingly, genes with proximal Smad2 binding without Eomesa are also significantly upregulated in MZ*eomesa* mutants, which may suggest that Eomesa has an indirect role in regulating Smad2-mediated transcriptional repression. Furthermore, these genes are highly enriched for chromatin assembly genes (see Additional file 8: Table S4), as identified previously (Figures 2F, 3E, 5C and Additional file 8: Table S4). This may suggest that Eomesa directly regulates genes which interact with Smad2 in the regulation of Eomesa-independent Smad2 targets.

Further analysis shows that genes downregulated in MZ*eomesa* mutants are associated with Nodal sites of action in zebrafish blastulae (Figure 5D; Additional file 8: Table S4), once again linking Eomesa with transcriptional activation in the context of Nodal signalling. On the other hand, genes that are upregulated in MZ*eomesa* mutants are associated with later embryonic neural structures (Figure 5E; Additional file 8: Table S4), supporting our earlier observation that genes with Eomesa proximal binding are involved in neurogenesis (Figure 3G, Additional file 8: Table S4), and suggesting that Eomesa acts potentially independently of Smad2 to repress expression of neurectodermal genes.

**Figure 6:**
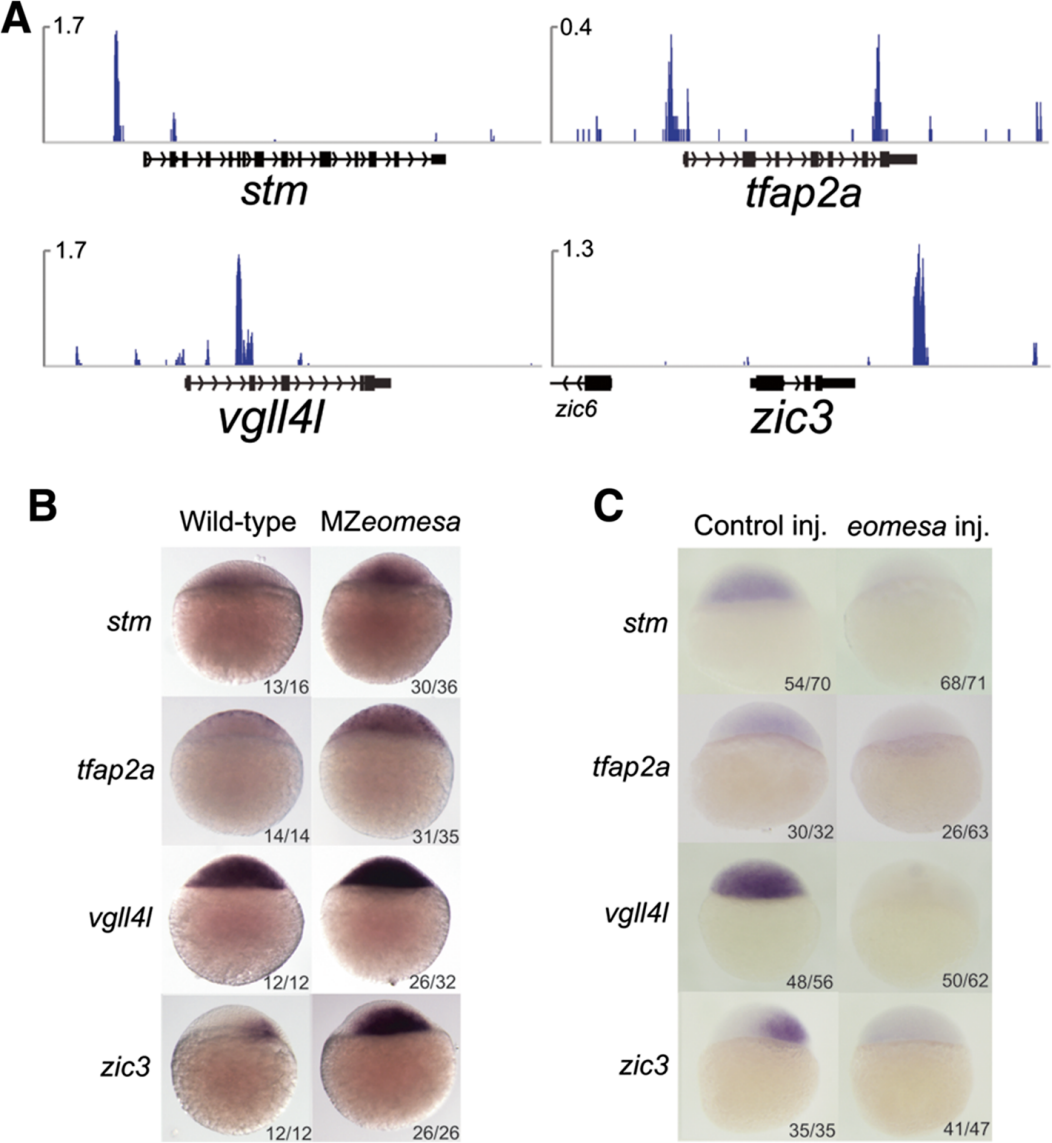
**Eomesa negatively regulates neural marker gene expression. (A)** ChIP-seq data showing binding of Eomesa at sphere stage proximal to neural marker genes; scale in reads per million reads. **(B)** *In situ* hybridisation of wild type and MZ*eomesa* mutant embryos for *stm*, *tfap2a*, *vgll4l* and *zic3* showing upregulation of these genes (lateral view, animal to the top). **(C)** *In situ* hybridisation of control injected and *eomesa* injected embryos for *stm*, *tfap2a*, *vgll4l* and *zic3* showing downregulation of these genes (lateral view, animal to the top). ChIP, chromatin immunoprecipitation sequencing.

In order to explore this further we picked four genes - *stm*, *tfap2a*, *vgll4l* and *zic3 -* that are upregulated in our MZ*eomesa* RNA-seq data and that exhibit proximal Eomesa binding (Figure 6A), and examined their expression by *in situ* hybridisation in MZ*eomesa* mutant blastulae and in wild-type embryos into which we had injected *eomesa* mRNA. Blastula expression of these genes has not been previously described, although later expression has. *stm*, for instance, is expressed in the anterior neural plate during gastrulation, whilst later in development, it is expressed in the otic vesicle [55,56]. *tfap2a* and *vgll4l* are expressed in the non-neural ectoderm during early gastrulation then become localized to the neural crest [57]. *zic3* is expressed at high levels in the neural plate during gastrulation as well as in the margin [58]. Our results show that these genes are upregulated in MZ*eomesa* mutants (Figure 6B), confirming the RNA-seq data, whilst overexpression of *eomesa* leads to strong repression of these genes (Figure 6C). These data confirm that Eomesa acts to repress transcription of genes normally expressed in the ectoderm.

## Discussion

### An evolutionarily conserved transcriptional network directed by Smad2 in mesendoderm gene expression

Nodal signalling is an absolute requirement for normal development in the early vertebrate embryo. In zebrafish Nodal signalling is active during blastula stages and is required throughout this period to specify mesoderm and endoderm tissues [35]. Whilst various studies have sought to identify Nodal targets in zebrafish at gastrula stages [17,34,59] our present study is the first to explore on a genome-wide scale the targets of Nodal at this critical time point of mid-blastula stage. Through this approach we identified both novel and known targets of Nodal signalling, shedding new light on the developmental mechanisms downstream of Nodal.

In order to identify direct targets of Nodal signalling in the zebrafish blastula we generated ChIP-seq data for Smad2. Whilst both Smad2 and Smad3 mediate Nodal target gene expression, genetic studies in mouse suggest that Smad2 is critical in early development whereas Smad3 is not [60-64]. Furthermore, our RNA-seq data indicate that *smad2* is 25-fold more highly expressed at sphere stage than either *smad3a/b*. From this we conclude that Smad2, rather than Smad3, is more likely to be the critical effector of Nodal signalling in early zebrafish development. We cannot discount the possibility, however, that Smad3 performs important roles downstream of Nodal during the developmental period covered in our study.

In assessing the relevance of model organisms, as well as in the study of evolutionary biology, the extent to which common phenotypes are achieved through common mechanisms is of great interest. Thus, we utilized Smad2 ChIP-seq datasets from mammalian *in vitro* experiments as a basis for comparison with a *Xenopus in vivo* Smad2 ChIP-seq dataset and our zebrafish dataset. This analysis revealed that the transcriptional network directed by Smad2 is broadly conserved. Our analyses indicate that genes with proximal Smad2 binding are more likely to be induced by Ndr1, to function in Nodal-mediated processes, and to be expressed at known sites of Nodal activity in zebrafish. This is a critical result as it implies that the mesendoderm development directed by Nodal occurs by more consistently conserved mechanisms than previously recognized. Our data also provide a valuable resource for identifying candidate genes involved in mesendoderm formation downstream of Nodal signalling.

### Interaction of Eomesa with Smad2 and Foxh1 in mesendoderm formation

Smad2 interacts with a range of other transcription factors to regulate transcription [2]. Our ChIP-seq datasets combined with our *ndr1* overexpression microarray data indicate that Smad2 and Eomesa co-bind genomic regions and positively regulate the expression of genes associated with the classical developmental functions of Nodal signalling in zebrafish. The interaction of Eomesdermin with Smad2 may be a general mechanism downstream of early Nodal signalling in vertebrates since in *Xenopus* Eomesdermin has been shown also to interact physically with Smad2 [18].

Smad2 alone binds upstream of chromatin assembly genes in zebrafish. This is likely to be biologically significant since our interspecies analysis indicates Smad2 binding proximal to orthologous genes in mouse. It has long-since been recognized that different levels of Nodal signalling leads to regulation of different genes, giving rise to different cell fates [2]. Regulation of chromatin assembly by Smad2 may relate to how dose-specific regulation of Nodal target genes is achieved as it may lead to alterations in the available *cis*-regulatory elements through which Smad2 acts. Eomesa does not bind proximal to these genes suggesting they are not direct transcriptional targets of Eomesa. Nevertheless, these genes are upregulated in MZ*eomesa* mutants, suggesting that Eomesa regulates these genes indirectly. Recently Gokhman *et al.* demonstrated that chromatin assembly genes are targets of TGFbeta-dependent Smad2 repression [42]. It is therefore possible that Eomesa regulates factors which cooperate with Smad2 to mediate this repression. Further study of Eomesa target gene function and Smad2 interacting factors will be required to determine this.

Our data, together with that of Slagle and colleagues [33], indicate that Eomesa and Foxh1 have combinatorial activities in regulating endodermal and mesodermal gene expression. Foxh1 contains a Smad2 interaction domain [15], but we do not know if Foxh1/Smad2 act in parallel to Eomesa/Smad2 downstream of Nodal signalling, or whether all three factors form a complex to regulate gene expression together. That genomic regions co-occupied by both Smad2 and Eomesa are more likely to contain a putative Foxh1 binding site than regions individually occupied by either factor suggests that at least in some cases the three factors act through the same regulatory elements. It may also be that in zebrafish much of Foxh1 genomic binding occurs through physical interaction with other transcription factors, rather than direct binding to DNA and, therefore, binds more regulatory elements than predicted in our study. In support of this, Slagle and colleagues demonstrated that *foxh1* mutants lacking the Smad interaction domain have a more severe phenotype than those lacking DNA binding ability [33]. Furthermore, in human SMAD2/3 and FOXH1 ChIP-seq data only around a quarter and a half of peaks, respectively, contain the canonical Foxh1 binding motif [19].

Our observations and conclusions on the relative contributions of Eomesa and Foxh1 to mesendodermal gene expression substantiate and further the observations made by Slagle *et al.* [33]. Their study used a dominant negative *eomesa* in the context of a *foxh1* mutant, and suggested that the two factors together regulate the entirety of Nodal signaling in early development. We now know, however, that the *eomesa* dominant negative and genetic null phenotypes are non-equivalent [25,26], and the use of dominant negative approaches with Tbox factors is increasingly considered unreliable due to the common DNA binding element shared by all factors [65]. Our *foxh1* knockdown in MZ*eomesa* mutants suggests that *eomesa* and *foxh1* do indeed combinatorially mediate endoderm formation and at least some early mesoderm marker expression. Furthermore, our data suggest that Eomesa and Foxh1 are at least partially functionally redundant, since overexpression of *foxh1* can partially rescue loss of *sox32* expression in MZ*eomesa* mutants. This redundancy is likely to occur through common regulatory elements used by both these factors and Smad2 downstream of Nodal. It is possible that more complete abrogation of Foxh1 function in MZ*eomesa* mutants would reveal further functional relationships and attempts to generate double maternal zygotic mutants for Eomesa and Foxh1 are ongoing.

Zebrafish contain two *Eomesodermin* homologues and it is possible that Eomesa acts redundantly with its paralogue as well as other factors. However, our data suggest that this is highly unlikely. In our RNA-seq experiment, although we detect expression for 78% of all annotated genes (25,361/32,468), we do not detect expression of *eomesb* either in wild-type or MZ*eomesa* mutant embryos. From this we conclude that *eomesa* and *eomesb* are not functionally redundant in early development.

### A role for Eomesa in ectodermal gene expression

Perhaps our most striking finding is that Eomesa acts to repress early ectodermal gene expression, suggesting that as well as positively influencing mesendodermal formation through activation of mesendodermal genes, it also exerts an influence through repressing inappropriate ectodermal gene expression at the onset of zygotic transcription.

Eomesa is expressed in the zebrafish telencephalon at later developmental stages [66] and a role for Eomes in neurogenesis has previously been revealed in mouse brain [67]. Significantly, our data indicate a role in the regulation of neuroectoderm gene expression far earlier than previously realized. Amongst the Eomesa-repressed genes are critical regulators of neurectodermal structures. For example, overexpression of *tfap2a* induces ectopic neural crest in zebrafish [68]; loss of Zic3 leads to broadened neural plate [69]. However, not all of the neuroectoderm-expressed genes we identify as being repressed by Eomesa are restricted to the neuroectoderm, for example, *sp5l*, *fgfr1b* and *zic3* are expressed in some mesodermal structures, such as the segmental plate. Nevertheless, anatomy term analysis shows that neuroectodermal expression, as based on published descriptions of expression at later stages than our study, is the defining characteristic of Eomesa-repressed genes. Our results represent a completely new component in the regulation of ectodermal gene expression in the early zebrafish embryo.

Eomesodermin is generally considered an activator of transcription [25], although it has recently been proposed to directly repress a subset of mesodermal genes during endoderm formation *in vitro* in human and in mouse embryos [23]. Eomesa binds upstream of 141 genes whose expression is up-regulated in the absence of Eomesa in zebrafish blastulas, suggesting it is directly repressing these genes. However, we are unable to distinguish whether Eomesa acts itself as a repressor or whether it interacts with another protein that represses gene expression. The latter possibility seems most likely given that in the early embryo Eomesa expression is not spatially restricted [25,26], it can both activate and repress gene expression ([25,26,28,70]; this study), and in activating mesendodermal gene expression it does so through interaction with Smad2. In this scenario the activity of Eomesa would depend on the partner it interacts with, allowing it to have different roles in different domains of the embryo if the partner protein had restricted expression or activity.

We note a recently published study by Gentsch and colleagues, which implicates Eomesodermin and other T-box factors in *Xenopus* in neural-mesodermal cell fate decisions [65]. In their study they find that combinatorial loss of T-box factors at gastrula and tail bud stages leads to a failure to specify mesoderm, with consequent gain of neural tissues. Our finding that Eomesa acts in transcriptional repression of ectodermal genes at the onset of zygotic transcription is fundamentally different; it demonstrates that in zebrafish a maternally contributed T-box factor restricts the expression of ectodermal genes at blastula stages. Combined with its known role in mesendoderm formation our analyses reveal that Eomesa regulates the formation of all three germ layers in the early embryo.

## Conclusions

In this study we investigated the transcriptional network directed by Nodal signalling in the early zebrafish embryo and its relationship with human, mouse and *Xenopus* data. We did this using a combination of Smad2 ChIP-seq and expression microarrays. Our data indicate that Smad2 directs highly similar transcriptional networks in several vertebrate species and provide a new insight into the evolutionarily conserved mechanisms of mesoderm and endoderm formation mediated by Nodal. Our data also represent a valuable resource for identifying potentially interesting genes for study in the context of Nodal signalling. Comparison of zebrafish Smad2 and Eomesa ChIP-seq data suggests that Eomesa acts as a general mediator of the conserved developmental functions of Nodal signalling in the early zebrafish embryo. Eomesa also acts to repress expression of ectoderm markers. As a maternally contributed factor Eomesa protein is present at the onset of zygotic transcription. Our data indicate that its early function is to activate a conserved Nodal transcriptional network whilst repressing ectodermal genes, thus ensuring correct spatiotemporal expression in early development.

## Methods

### Ethics statement

All zebrafish studies complied fully with the UK Animals (Scientific Procedures) Act 1986 as implemented by King’s College London or were in accordance with the policies of the University of Toronto Animal Care Committee.

### Zebrafish strains

AB and mutant zebrafish were reared as described [71]. MZ*oep*^*m134*^ embryos were used in this study. MZ*eomesa*^*fh105*^ embryos were generated by *in vitro* fertilization using homozygous mutant parents. In some experiments M*eomesa*^*fh105*^ embryos were generated by *in vitro* fertilization using homozygous mutant female and heterozygous or wildtype male parents and used in place of MZ*eomesa* embryos. In this and previous studies [26,46] no differences in endodermal or mesodermal gene expression at blastula stages were seen between MZ*eomesa* or M*eomesa* embryos.

### mRNA, morpholinos and microinjections

Capped mRNA was synthesized from pCS2+ eomesa, pCS2+ eomesa-eng, CS2+ ndr1 and CS2 + foxh1 as described [7,25,33]. As a control, *egfp* mRNA was used. One-cell stage embryos were injected with 10 pg *ndr1*, 400 pg *Eomesa* mRNA, 20 pg *foxh1* mRNA or the same amount of *gfp* mRNA. One-cell stage embryos were injected with 2.5 ng of a Foxh1 translation blocking morpholino (5′ CCAGTGCTTTGTCATGCTGATGTAG). We also tested a previously published morpholino (5′ TGCTTTGTCATGCTGATGTAGTGGG) [72] and found it gave the same phenotype as ours at the same injection concentration, but with less penetrance. We, therefore, used our morpholino in this study.

### Antibodies and validation

For Smad2 ChIP we used a rabbit monoclonal antibody (clone 31H15L4; Thermo Fisher Scientific, Waltham, MA, USA) which is raised to amino acids 81 to 107 of human Smad2 (Q15769). This sequence maps to amino acids 82 to 108 of zebrafish Smad2 (AAF06737; Figure S2A, see Additional file 3), but does not map to zebrafish Smad3. ChIP-western blotting shows that this antibody recognizes Smad2 (Figure S2B, see Additional file 3).

For Eomesodermin ChIP we used a polyclonal antibody that was raised against the full-length zebrafish Eomesodermin protein (Absea, Beijing, China). Because the T-box is a conserved structure, it is possible that the antibody could cross-react with the T-box of related proteins, so we sought to test this. RNA-seq data showed that in high stage embryos Ntla, Tbx16 and Mga are also expressed at high levels (>10 FPKM; Figure S2C, see Additional file 3). Ntla has 50.5% identity across the T-box, while Tbx16 has 48.6% identity and Mga has 42.6% identity. We tested the specificity of the anti-Eomesa antibody using *in vitro* translated Eomesa, Ntla and Tbx16 [73] and found that the antibody specifically recognizes Eomesa and does not significantly cross react with Tbx16 or Ntla (Figure S2D, see Additional file 3). ChIP-western blotting shows that this antibody recognizes Eomesodermin (Figure S2E, see Additional file 3).

As a secondary validation [43] we used *de novo* motif searching under ChIP-seq peaks to validate the antibodies and were able to detect enrichment of the Smad binding element in Smad2 bound regions and the T-box binding site in Eomesa bound regions (see Results).

### *In vitro* protein production and immunoprecipitation

^35^S-labeled protein for Ntla, Tbx16 and Eomesa was synthesized in reticulocyte lysate as previously described [74]. Proteins were immunoprecipitated using anti-Eomesa antibody and the eluate subjected to SDS/PAGE. Equivalent input reticulocyte lysates were also subjected to SDS/PAGE for comparison. Gels were fixed and dried then exposed to X-ray film to detect ^35^S-radiolabelled proteins (Figure S2D, see Additional file 3).

### ChIP-western blots

Embryos were injected with 400 pg of Smad2 mRNA or Eomesodermin mRNA. A total of 100 embryos (injected or uninjected) were then processed as if for ChIP with either anti-Smad2 antibody (0.25 μg), anti-Eomesa antibody (1 ul) or a no antibody control. The immnuoprecipitated proteins were then eluted from the beads and subjected to SDS/PAGE. Western blotting was then performed with anti-Smad2 antibody (1:2,000) or anti-Eomesa antibody (1:600) then protein A-HRP (1:20,000; Merck Millipore, Billerica, MA, USA) or anti-Rabbit IgG-HRP (1:20,000; Thermo Fisher Scientific, Waltham, MA, USA).

### Chromatin immunoprecipitation

For ChIP-seq, two independent reactions were performed on 5,000 high-sphere embryos as described [73,75,76] except that a cleavable oligo used in the ligation-mediated PCR which was then removed after amplification. Either anti-Eomesa (50 μl) or anti-SMAD2 (10 μg) antibody were used. Illumina paired-end libraries were prepared according to the manufacturer’s instructions, size selected and sequenced on the Illumina GAIIx platform (Illumina, San Diego, CA, USA).

### ChIP-qPCR

ChIP-qPCR was performed on approximately 300 to 500 embryos at high-sphere stage using 3 μl anti-Eomesa serum, 0.5 μg anti-Smad2 antibody and normal rabbit IgG. Peaks with high, medium or low heights were picked for validation. For Smad2 peaks low is ≤1.5 reads per million (RPM); medium is 1.5 to 3 RPM; high is >3 RPM. For Eomesa low is ≤1 RPM; medium is 1 to 1.5 RPM; high is >1.5 RPM. Eomesa have lower RPM scores as reads are split between more peaks. In addition, a negative region (upstream of *rhodopsin* [73]) was included as a comparison. qPCR was carried out on a Mx3005P (Agilent Technologies, Santa Clara, CA, USA) using SYBR Green 1 Master kit (Roche Basel, Switzerland) according to the manufacturer’s instructions and at 60°C annealing temperature. Values for each condition were calculated as a percentage of input and are presented as fold enrichment relative to the IgG control. Primer sequences were as follows: Smad2 peak 10: forward - 5′ TTCTCCTCCTGCACCTTCTG, reverse - 5′ GGGGATGAAGAGTCTCTGGG; Eomesa peak 2624: forward - 5′ GCCAATTAGCATGTGTGGCAATT, reverse - 5′ CCTGTTGCTCTGGTGTGACAC; Smad2 peak 793: forward - 5′ TGCCCTGTAAGAGCACTACA, reverse - 5′ GGGCTACTGTGGCTTAGTC, Eomesa peak 5571: forward - 5′ CGCTCCGCCATCACTTTAAA, reverse - 5′ TTTCCTTCGGCAACTTTCGG; Smad2 peak 796: forward - 5′ TGGCTCTCCTCGTTCTTCTGT, reverse - 5′ AGTCAAATCCGAGTGCCCATGA; Common peak 54: forward - 5′ TGATGAGCTGCAGGATAACG, reverse - 5′ GAGTCTGTCTGGCGCTCTCT; Common peak 72: forward - 5′ CCGGGTGTGAATTAGCATCT, reverse - 5′ TGCTACAGTCGGCAAACATC; Common peak 156: forward - 5′ TTTACTGGGACGGCCATTAG, reverse - 5′ GCTCATCAGGCTGGAGTCAT; Common peak 187: forward - 5′ ATTAAACTCGCACACGAACCTT, reverse - 5′ CCTGAAATGAGTGGCTTTTCTT; Eomesa peak 3484: forward - 5′ TCTGACACCTCACACATGCA, reverse - 5′ GCAGATTTGGGAGTTCAGCC.

### Defining Transcription Factor binding events

All reads were converted to Sanger FASTQ format and mapped to the Zv9 version of the zebrafish genome using Bowtie [77] in Galaxy [78-80]. Alignments were performed using the following criteria: -n2 –e120 –l 28 -m2 -k2 -best. Peak calling, relative to paired input samples (whole chromatin), was subsequently performed using MACS [81] using default parameters except as follows: −−mfold8 –pvalue 1e-4. Overlapping peaks from both independent ChIP-seq experiments for each factor were identified and used for further analysis. Parameters were chosen to take into account peak proximity to genes, including Nodal-responsive genes and known targets, and concordance with known targets of Nodal signaling, experimental reproducibility and enrichment for the known binding motifs. Peaks were visualized using Integrated Genomics Viewer (IGV) [82,83].

### Motif analysis

*De novo* motif finding was performed with Weeder v. 1.4.2 using default settings [84]. Best matches to the Smad binding element and Tbox motifs identified by Weeder, and Foxh1 binding sites (JASPAR motif MA0479.1) for each ChIP-seq peak were identified using Perl scripts modified from the transcription factor binding site (TFBS) suite [85]. Enrichment of Foxh1 binding sites between peak sets was determined using Chi-square test.

### RNA extraction and microarray hybridisation

Total RNA was extracted from sphere stage embryos using Trizol (Invitrogen) according to the manufacturer’s protocol. RNA samples were treated with RQ Dnase I (Promega, Madison, WI, USA) and cleanup performed using Rneasy Mini Kit (QIAGEN, Hilden, Germany). A total of 15 μg of total RNA was used for Alexa Fluor® 555-labelled cDNA production using the SuperScript™ Plus Direct cDNA Labeling System (Invitrogen). RNA was then hydrolysed through addition of NaOH and heating to 70°C and subsequently neutralised with HCl. The reaction was then cleaned using the PureLink PCR Purification Kit (Invitrogen). Hybridisation cocktails were produced and hybridised to Agilent Zebrafish Gene Expression Microarrays (V2), washed and dried according to the manufacturer’s instructions. Microarrays were scanned and image analysis performed using an Axon GenePix 4000B scanner and software.

### Microarray data analysis

In order to make microarray data appropriately comparable with ChIP-seq data all GenBank IDs to which microarray probes were designed were mapped to cDNAs in the Ensembl release 64. Microarray data were qspline normalised and differential expression determined using the R package oneChannelGUI [86]. Association with ChIP-seq identified genes was inferred by the Chi-square test.

### Identification of conserved transcriptional networks

ChIP-seq peak coordinates were downloaded from GEO accessions GSE23581 [40], GSE29422 [39] and GSE30146 [19]. Genes of Ensembl release 64 with Smad2 binding within 10 kb of transcription start sites in each species were then converted to zebrafish orthlogue and possible orthologues defined in Ensembl release 64. The lists of zebrafish genes derived from each species were compared. Functional and anatomical analysis of the resulting lists was performed using DAVID Bioinformatics Resource 6.7 [87,88]. Association between genes with proximal Smad2 binding and Ndr1-responsive genes was inferred by the Chi-square test. Reciprocal analysis was also performed by converting zebrafish Ndr1-responsive genes to orthologues and possible orthologues in mouse, human and *Xenopus*. These genes were then compared to genes with proximal Smad2 binding in each species by the Chi-square test.

### RNA extraction and RNA-seq analysis

Total RNA was isolated from sphere stage wild type and MZ*eomesa* embryos using Trizol (Invitrogen) according to the manufacturer’s instructions. DNA was removed using the TURBO DNA-free kit (Invitrogen). The Johns Hopkins Deep Sequencing and Microarray Core Facility constructed the RNA-seq libraries and performed single-read 100 bp read length sequencing on an Illumina HiSeq 2000. The facility mapped the reads using Tophat 2 [89] with the options -p 8 -i 20 -I 380000 --coverage-search --microexon-search for each sample. Differential expression was calculated using Cufflinks [90] with options –p 4 -u.

### In situ hybridisations

*In situ* hybridisations were performed as described [91]. Anti-sense riboprobes for *gsc* [92]; *ntla* [93]; *ttna* [94], *sox32* [53], *stm* [55], *tfap2a* [57] and *zic3* [58] were generated as described. *klf3*, *foxa*, *notum1a*, *nhsl1b*, *smarcd3b* and *vgll4l* were amplified from 24 hpf or mixed stage cDNA, cloned into pGEM-Teasy and antisense riboprobes generated by standard methods. The primers to clone these probes are detailed below:

- *Foxa* forward – 5′ TCTCCAGACTCTCCAGGAAAAG; reverse – 5′ TCCAAAACATTCACAGAACCAC.
- *Klf3* forward – 5′ CAAAAGTAGCTTTCGAGCACCT; reverse – 5′ GCTCTCTATAAATTGCCCATTCAG.
- *Notum1a* forward - 5′ GACCACCGAGAGCTTCTCAC; reverse – 5′ GGATCCATTAACCCTCACTAAAGGGACTGACCTGGATGTTGGTGTG
- *Nhsl1a* forward – 5′ GCTGGAAAAAGAGGAAGAGACA; reverse -5′ GCCCAGAAGACCTTATTCCTTT.
- *Smarcd3b* forward – 5′ TATATGGATCTGTTGGCATTCG; reverse – 5′ CCTGTCTCCTCTGCTGAATCTT.
- *Vgll4l* forward - 5′ ATGGCGGTCACTAATTTCCACTA; reverse – TCATTTATCAGACCAGAAGTTTG.

### Accession numbers

Primary microarray, ChIP-seq and RNA-seq data have been deposited in the National Center for Biotechnology Information (NCBI) Gene Expression Omnibus under accession number GSE51894.

## Additional files

Additional file 1: Table S1. Genes differentially expressed (*P* ≤0.02) in the *ndr1* overexpression microarray experiment. Additional file 2: Figure S1. Targets of Nodal signalling.**(A)** Venn diagram indicating the overlap between *ndr1*-responsive genes identified in this study and Nodal target genes identified in previous studies. **(B)** *In situ* hybridisation of wild type, *ndr1* mRNA-injected and MZ*oep* mutant embryos for *klf3*, *nhsl1b* and *notum* at sphere showing up regulation in response to *ndr1* mRNA injection and downregulation of expression in MZ*oep* embryos, which lack Nodal signalling. Lateral views. Numbers on each panel indicate the number of embryos showing the phenotype depicted over the total number of embryos analysed. Additional file 3: Figure S2. Anti-Smad2 and anti-Eomesa antibodies are specific. **(A)** Alignment of the peptide immunogen (from human Smad2) used to generate the anti-Smad2 antibody with zebrafish Smad2 protein sequence. Zebrafish Smad2 has 81.5% identity with the peptide across the aligned region. **(B)** ChIP-western on embryos overexpressing Smad2 detects a band at 60 KDa corresponding to zebrafish Smad2 (lane 2). The same size band is detected in uninjected embryos (lane 4) indicating the antibody detects endogenous Smad2. **(C)** Expression levels of all genes encoding transcription factors with T-box binding domains at sphere stage detected by RNA-seq (FPKM – fragments per kilobase of exon per million mapped reads). **(D)** The anti-Eomesa antibody immunoprecipitates *in vitro* translated Eomesa in reticulocyte lysates, but not Ntla and Tbx16. **(E)** ChIP-western on embryos overexpressing Eomesa detects a band at 76 KDa corresponding to zebrafish Eomesa (lane 2). Unlike for Smad2 we were unable to detect endogenous Eomesa by this method, suggesting Eomesa is expressed at lower levels in the embryo. Bands at 50 KDa correspond to IgG heavy chain from ChIP step. Additional file 4: Table S2. Genomic coordinates of Smad2 ChIP-seq peaks. Additional file 5: Figure S3. Validation of ChIP-seq target peaks. **(A)** qPCR-ChIP validation of Smad2 binding and **(B)** Eomesa binding to the indicated genomic regions identified in ChIP-seq experiments [see Additional files 4, 10 and 12] compared to a negative control region (upstream of *rhod*). Values are shown relative to the IgG control ChIP for three biological replicates. The categories into which the validated ChIP-seq peaks fall are - for Smad2 LLMMMHH; Eomesa LLMHHMM (L = Low; M = Medium; H = High, see Methods for further details). Additional file 6: Figure S4. Smad2 binding is enriched within 10 kb of transcription start sites of *ndr1*-responsive genes. **(A)** Distribution of Smad2 ChIP-seq peaks compared to random genomic regions relative to transcription start sites. †† *P* ≤4 × 10^−3^; ††† *P* ≤2 × 10^−9^. **(B)** Association of Smad2 binding at increasing distances from TSSs with *ndr1*-responsive genes. **P* ≤1 × 10^−2^; ***P* ≤4 × 10^−7^; ****P* ≤1 × 10^−18^. Additional file 7: Table S3. Ensembl genes represented as zebrafish orthologues with Smad2 binding ±10 kb of their transcription start sites in one or more species. Additional file 8: Table S4. Functional and anatomical enrichment results obtained using DAVID. Additional file 9: Figure S5. Sequence analysis of Smad2 and Eomesa ChIP-seq peaks reveals central enrichment for known bindings elements and significant enriched for the Foxh1 binding element amongst common Smad2/Eomesa ChIP-seq peaks. **(A)** Best single matches to the Smad binding element (SBE) and Tbox motifs identified in this study were identified for each ChIP-seq peak and their distribution plotted relative to peak centres. Binding elements are clearly most enriched towards the centre of peaks. **(B)** Doughnut plots showing the percentage of ChIP-seq peaks containing the SBE, Tbox (as defined by this study) or Foxh1 binding element (JASPAR Matrix ID MA0479.1), and the net motif representing all occurrences of each element used in this analysis. The percentage of common Smad2/Eomesa ChIP-seq peaks containing a Foxh1 binding elements is significantly greater than for either total Eomesa or total Smad2 peaks (*P* value =5.7 × 10^−30^ and 4.9 × 10^−3^ respectively). Additional file 10: Table S5. Genomic coordinates of Eomesa ChIP-seq peaks. Additional file 11: Table S6. Genomic coordinates of overlap of Eomesa and Smad2 ChIP-seq peaks, referred to as ‘common peaks’. Additional file 12: Figure S6. Eomesa and Foxh1 are required for robust sox32 expression. *In situ* hybridisation of *ntla* **(A-C)**, *gsc* **(D-F)** and *sox32* **(G-K)** at early shield stage in wild-type embryos, MZ*eomesa* mutants and MZ*eomesa*;*foxh1* morphants. Compared to 30% epiboly (Figure 4) *ntla* expression in MZ*eomesa*;*foxh1* morphants, recovers dorsally and to varying degrees in the ventral and lateral margin with 6/10 showing absence (shown in C) or decrease in the lateral margin and 2/10 showing absence in the ventral margin. *gsc* expression in MZ*eomesa*;*foxh1* morphants, on the other hand, recovers to look similar to MZ*eomesa* mutants by shield stage. By shield stage *sox32* expression in the blastoderm of MZ*eomesa* mutants has recovered to a variable extent in the ventral and lateral margin, although not the ventral-lateral YSL with some embryos showing little recovery (H) and others showing more (J). In MZ*eomesa*;*foxh1* morphants dorsal expression in the YSL and blastoderm has recovered to some extent, but little (K) or almost no expression (I) is seen in the ventral or lateral margin. Animal views; dorsal to the right. Numbers on each panel indicate the number of embryos showing a phenotype over the total number of embryos analysed. Additional file 13: Table S7. Genes differentially expressed (*P* ≤0.05) between wild-type and MZeomesa sphere stage embryos in the RNA-seq experiment.
