# Supplementary figures and images for: Global identification of Smad2 and Eomesodermin targets in zebrafish identifies a conserved transcriptional network in mesendoderm and a novel role for Eomesodermin in repression of ectodermal gene expression
Source: BMC Biol. 2014 Oct 3;12:81. doi: 10.1186/s12915-014-0081-5 (PMC4206766; doi:10.1186/s12915-014-0081-5)

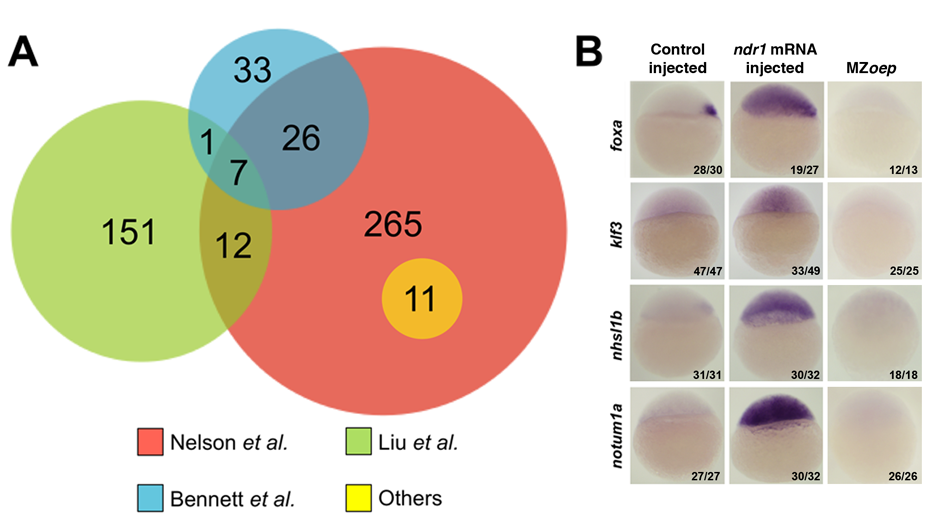

Supplement: Additional file 2: Figure S1. — Targets of Nodal signalling.(A) Venn diagram indicating the overlap between ndr1-responsive genes identified in this study and Nodal target genes identified in previous studies. (B) In situ hybridisation of wild type, ndr1 mRNA-injected and MZoep mutant embryos for klf3, nhsl1b and notum at sphere showing up regulation in response to ndr1 mRNA injection and downregulation of expression in MZoep embryos, which lack Nodal signalling. Lateral views. Numbers on each panel indicate the number of embryos showing the phenotype depicted over the total number of embryos analysed. [file 12915_2014_81_MOESM2_ESM.tiff]

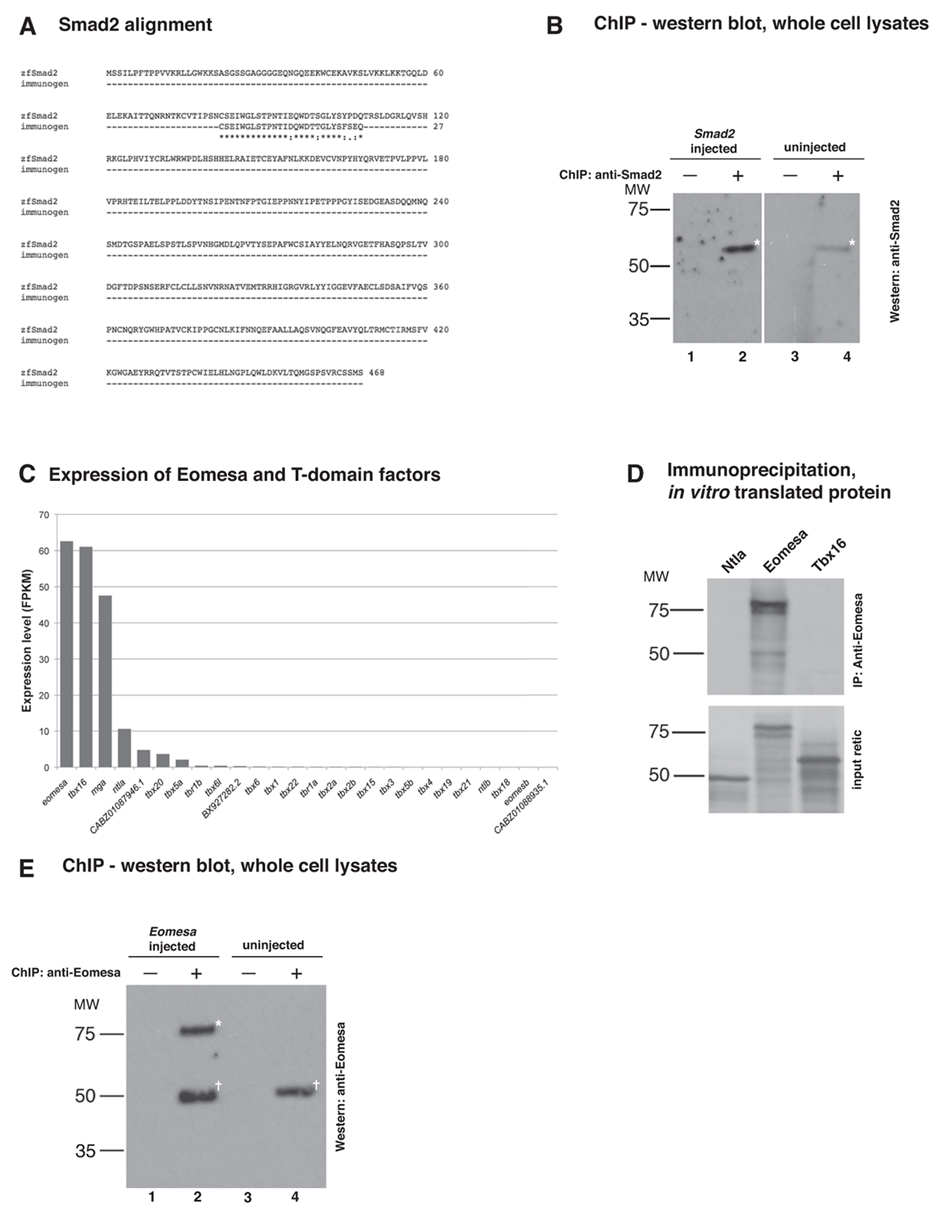

Supplement: Additional file 3: Figure S2. — Anti-Smad2 and anti-Eomesa antibodies are specific. (A) Alignment of the peptide immunogen (from human Smad2) used to generate the anti-Smad2 antibody with zebrafish Smad2 protein sequence. Zebrafish Smad2 has 81.5% identity with the peptide across the aligned region. (B) ChIP-western on embryos overexpressing Smad2 detects a band at 60 KDa corresponding to zebrafish Smad2 (lane 2). The same size band is detected in uninjected embryos (lane 4) indicating the antibody detects endogenous Smad2. (C) Expression levels of all genes encoding transcription factors with T-box binding domains at sphere stage detected by RNA-seq (FPKM – fragments per kilobase of exon per million mapped reads). (D) The anti-Eomesa antibody immunoprecipitates in vitro translated Eomesa in reticulocyte lysates, but not Ntla and Tbx16. (E) ChIP-western on embryos overexpressing Eomesa detects a band at 76 KDa corresponding to zebrafish Eomesa (lane 2). Unlike for Smad2 we were unable to detect endogenous Eomesa by this method, suggesting Eomesa is expressed at lower levels in the embryo. Bands at 50 KDa correspond to IgG heavy chain from ChIP step. [file 12915_2014_81_MOESM3_ESM.tiff]

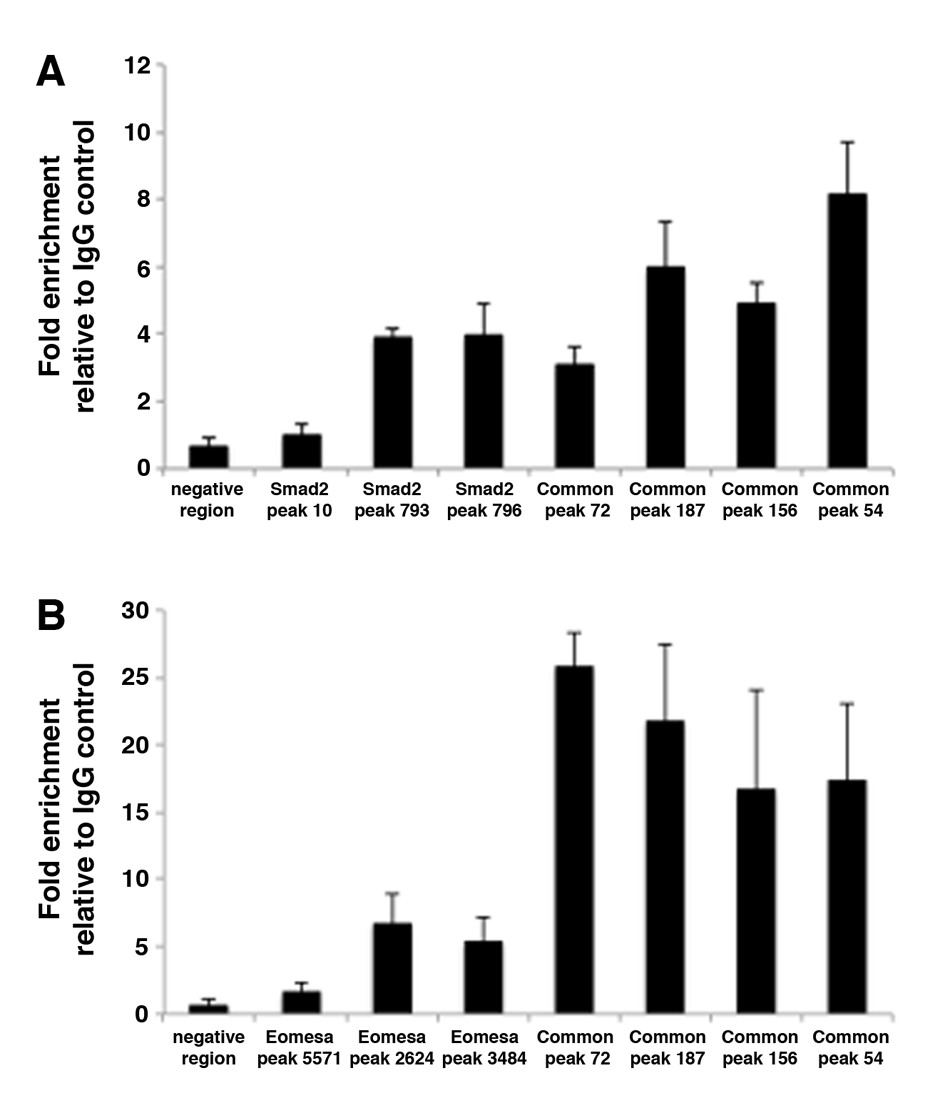

Supplement: Additional file 5: Figure S3. — Validation of ChIP-seq target peaks. (A) qPCR-ChIP validation of Smad2 binding and (B) Eomesa binding to the indicated genomic regions identified in ChIP-seq experiments [see Additional files 4, 10 and 12] compared to a negative control region (upstream of rhod). Values are shown relative to the IgG control ChIP for three biological replicates. The categories into which the validated ChIP-seq peaks fall are - for Smad2 LLMMMHH; Eomesa LLMHHMM (L = Low; M = Medium; H = High, see Methods for further details). [file 12915_2014_81_MOESM5_ESM.tiff]

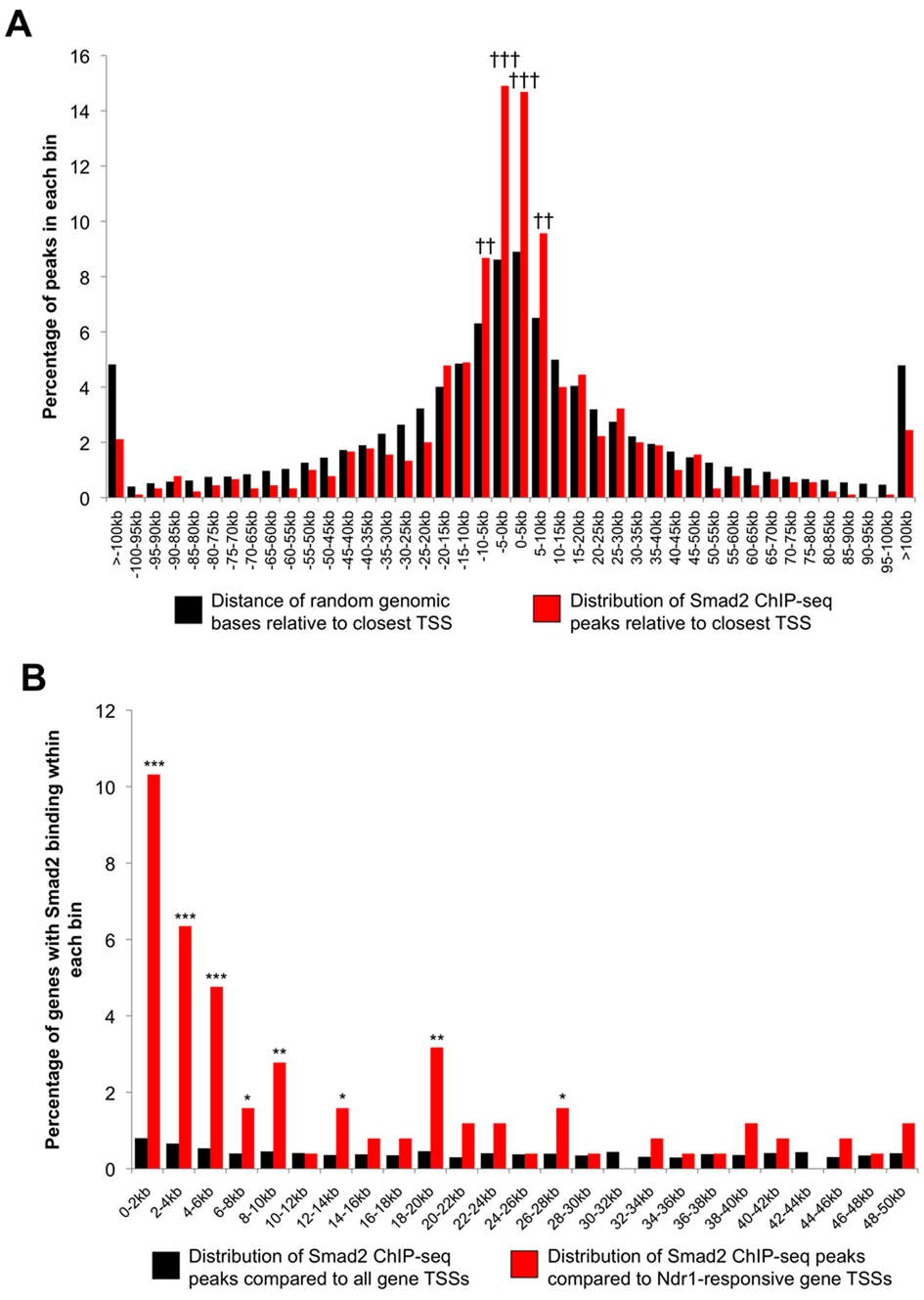

Supplement: Additional file 6: Figure S4. — Smad2 binding is enriched within 10 kb of transcription start sites of ndr1-responsive genes. (A) Distribution of Smad2 ChIP-seq peaks compared to random genomic regions relative to transcription start sites. †† P ≤4 × 10^−3^; ††† P ≤2 × 10^−9^. (B) Association of Smad2 binding at increasing distances from TSSs with ndr1-responsive genes. *P ≤1 × 10^−2^; **P ≤4 × 10^−7^; ***P ≤1 × 10^−18^. [file 12915_2014_81_MOESM6_ESM.tiff]

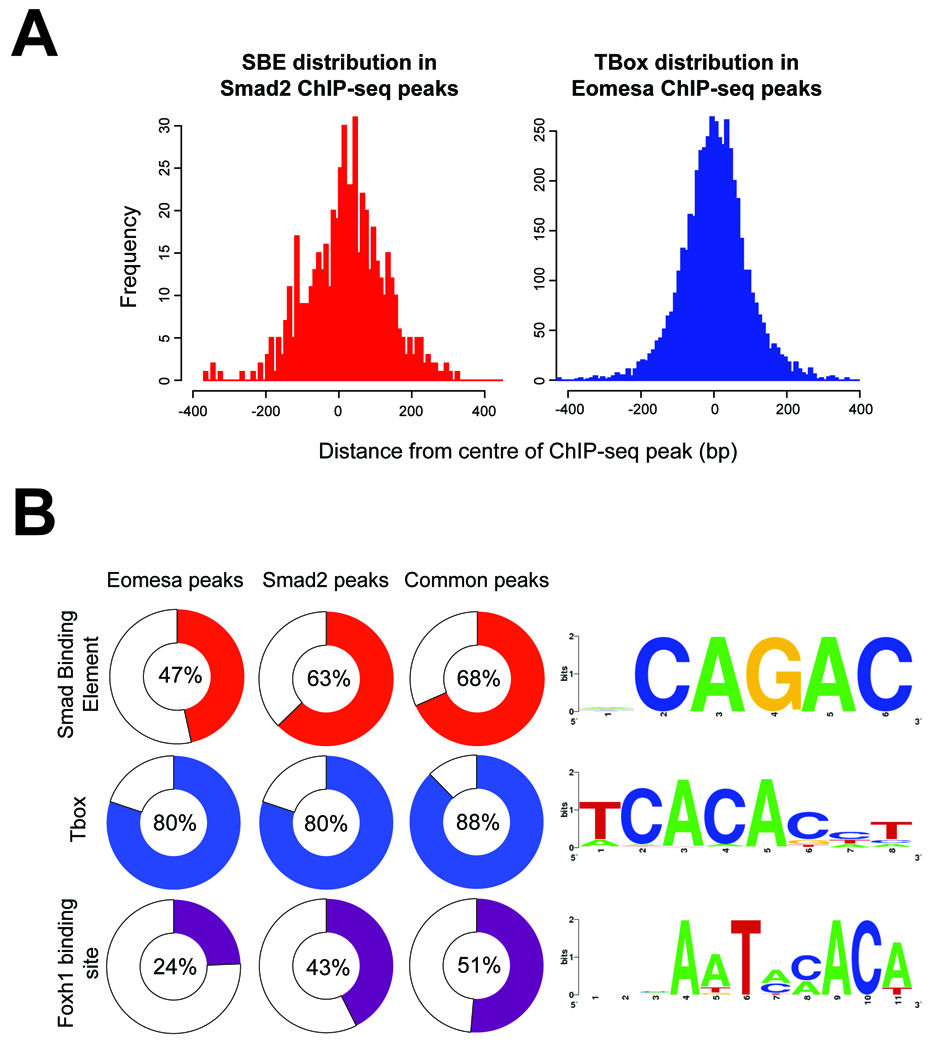

Supplement: Additional file 9: Figure S5. — Sequence analysis of Smad2 and Eomesa ChIP-seq peaks reveals central enrichment for known bindings elements and significant enriched for the Foxh1 binding element amongst common Smad2/Eomesa ChIP-seq peaks. (A) Best single matches to the Smad binding element (SBE) and Tbox motifs identified in this study were identified for each ChIP-seq peak and their distribution plotted relative to peak centres. Binding elements are clearly most enriched towards the centre of peaks. (B) Doughnut plots showing the percentage of ChIP-seq peaks containing the SBE, Tbox (as defined by this study) or Foxh1 binding element (JASPAR Matrix ID MA0479.1), and the net motif representing all occurrences of each element used in this analysis. The percentage of common Smad2/Eomesa ChIP-seq peaks containing a Foxh1 binding elements is significantly greater than for either total Eomesa or total Smad2 peaks (P value =5.7 × 10^−30^ and 4.9 × 10^−3^ respectively). [file 12915_2014_81_MOESM9_ESM.tiff]

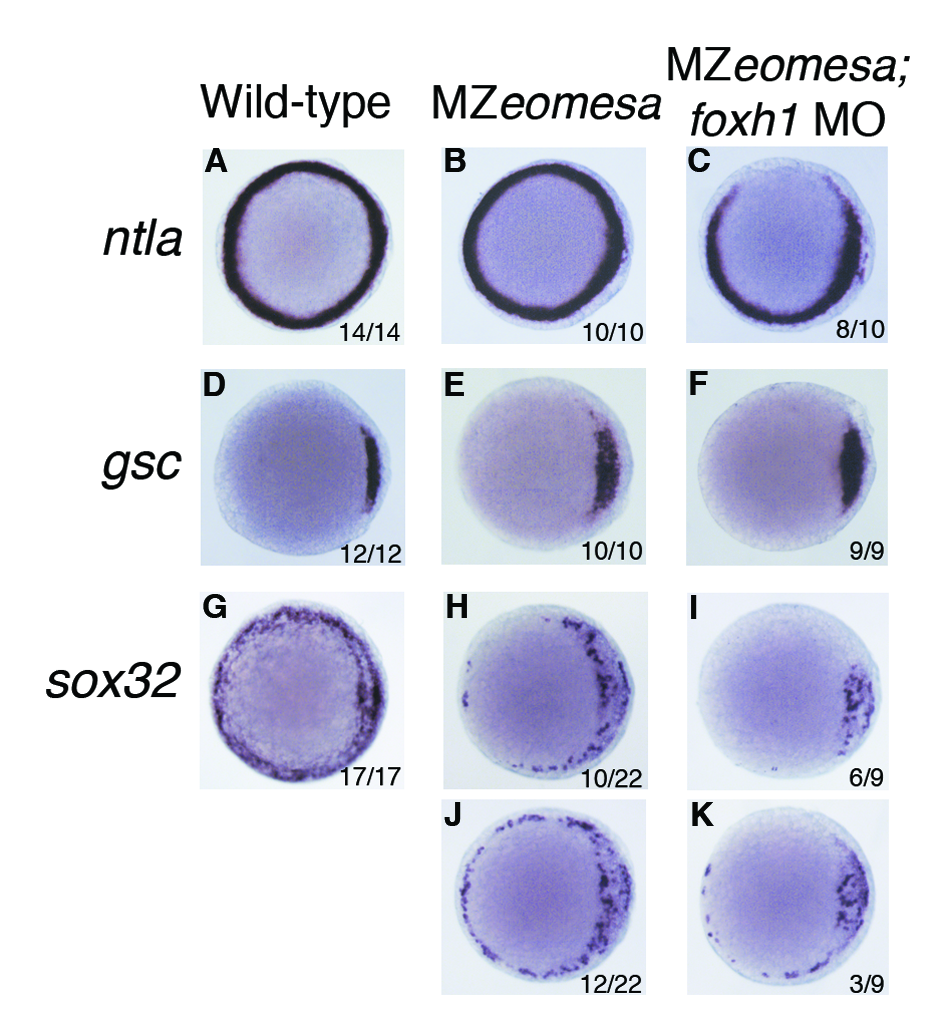

Supplement: Additional file 12: Figure S6. — Eomesa and Foxh1 are required for robust sox32 expression. In situ hybridisation of ntla (A-C), gsc (D-F) and sox32 (G-K) at early shield stage in wild-type embryos, MZeomesa mutants and MZeomesa;foxh1 morphants. Compared to 30% epiboly (Figure 4) ntla expression in MZeomesa;foxh1 morphants, recovers dorsally and to varying degrees in the ventral and lateral margin with 6/10 showing absence (shown in C) or decrease in the lateral margin and 2/10 showing absence in the ventral margin. gsc expression in MZeomesa;foxh1 morphants, on the other hand, recovers to look similar to MZeomesa mutants by shield stage. By shield stage sox32 expression in the blastoderm of MZeomesa mutants has recovered to a variable extent in the ventral and lateral margin, although not the ventral-lateral YSL with some embryos showing little recovery (H) and others showing more (J). In MZeomesa;foxh1 morphants dorsal expression in the YSL and blastoderm has recovered to some extent, but little (K) or almost no expression (I) is seen in the ventral or lateral margin. Animal views; dorsal to the right. Numbers on each panel indicate the number of embryos showing a phenotype over the total number of embryos analysed. [file 12915_2014_81_MOESM12_ESM.tif]
